# A Complex Genetic Switch Involving Overlapping Divergent Promoters and DNA Looping Regulates Expression of Conjugation Genes of a Gram-positive Plasmid

**DOI:** 10.1371/journal.pgen.1004733

**Published:** 2014-10-23

**Authors:** Gayetri Ramachandran, Praveen K. Singh, Juan Roman Luque-Ortega, Luis Yuste, Carlos Alfonso, Fernando Rojo, Ling J. Wu, Wilfried J. J. Meijer

**Affiliations:** 1 Centro de Biología Molecular “Severo Ochoa” (CSIC-UAM), Instituto de Biología Molecular “Eladio Viñuela” (CSIC), Universidad Autónoma, Canto Blanco, Madrid, Spain; 2 Centro de Investigaciones Biológicas (CSIC), Madrid, Spain; 3 Centro Nacional de Biotecnología (CSIC), Canto Blanco, Madrid, Spain; 4 Centre for Bacterial Cell Biology, Institute for Cell and Molecular Biosciences, Newcastle University, Newcastle Upon Tyne, United Kingdom; The University of Texas Health Science Center at Houston, United States of America

## Abstract

Plasmid conjugation plays a significant role in the dissemination of antibiotic resistance and pathogenicity determinants. Understanding how conjugation is regulated is important to gain insights into these features. Little is known about regulation of conjugation systems present on plasmids from Gram-positive bacteria. pLS20 is a native conjugative plasmid from the Gram-positive bacterium *Bacillus subtilis*. Recently the key players that repress and activate pLS20 conjugation have been identified. Here we studied in detail the molecular mechanism regulating the pLS20 conjugation genes using both *in vivo* and *in vitro* approaches. Our results show that conjugation is subject to the control of a complex genetic switch where at least three levels of regulation are integrated. The first of the three layers involves overlapping divergent promoters of different strengths regulating expression of the conjugation genes and the key transcriptional regulator Rco_LS20_. The second layer involves a triple function of Rco_LS20_ being a repressor of the main conjugation promoter and an activator and repressor of its own promoter at low and high concentrations, respectively. The third level of regulation concerns formation of a DNA loop mediated by simultaneous binding of tetrameric Rco_LS20_ to two operators, one of which overlaps with the divergent promoters. The combination of these three layers of regulation in the same switch allows the main conjugation promoter to be tightly repressed during conditions unfavorable to conjugation while maintaining the sensitivity to accurately switch on the conjugation genes when appropriate conditions occur. The implications of the regulatory switch and comparison with other genetic switches involving DNA looping are discussed.

## Author Summary

Plasmids are extrachromosomal, autonomously replicating units that are harbored by many bacteria. Many plasmids encode transfer function allowing them to be transferred into plasmid-free bacteria by a process named conjugation. Since many of them also carry antibiotic resistance genes, plasmid-mediated conjugation is a major mechanism in the dissemination of antibiotic resistance. In depth knowledge on the regulation of conjugation genes is a prerequisite to design measures interfering with the spread of antibiotic resistance. pLS20 is a conjugative plasmid of the soil bacterium *Bacillus subtilis*, which is also a gut commensal in animals and humans. Here we describe in detail the molecular mechanism by which the key transcriptional regulator tightly represses the conjugation genes during conditions unfavorable to conjugation without compromising the ability to switch on accurately the conjugation genes when appropriate. We found that conjugation is subject to the control of a unique genetic switch where at least three levels of regulation are integrated. The first level involves overlapping divergent promoters of different strengths. The second layer involves a triple function of the transcriptional regulator. And the third level of regulation concerns formation of a DNA loop mediated by the transcriptional regulator.

## Introduction

Bacteria exchange genetic material at high rates by different processes, which are collectively named Horizontal Gene Transfer (HGT). HGT can be beneficial for bacteria because the newly acquired DNA may endow them with novel features enabling them to adapt to changing conditions in the environment, i.e. rapid evolution. On the other hand, HGT is notorious for its role in the dissemination of virulence/pathogenicity determinants and antibiotic resistance. The main mechanisms responsible for HGT are transformation mediated by natural competence, transduction and conjugation [1]–[6]. The latter mechanism, -conjugation-, concerns the transfer of a DNA element from a donor to a recipient cell. Conjugative elements containing all the information required for DNA transfer of a donor to a recipient cell are often found on plasmids, but they can also be embedded within a bacterial chromosome. These latter forms are generally named integrative and conjugative elements (ICE).

Some basic features of the conjugation process are conserved among plasmids [for review see], [ 7]–[10]. In most cases, a single-stranded DNA (ssDNA), which is generated by a rolling circle-like mode of DNA replication, is transferred into the recipient cell through a membrane-associated intercellular mating channel, named transferosome, which is a form of type IV secretion system. Conjugative plasmids can be exploited for the construction of tools to genetically modify bacteria of clinical or industrial relevance that are reluctant to genetic manipulation by other ways. Besides its intrinsic scientific interest, a detailed understanding about how conjugation genes are regulated is crucial to design strategies helping to interfere with the rapid spread of antibiotic resistance, and for the construction of genetic tools based upon conjugative plasmids.

Various conjugative plasmids have been studied in considerable detail [for review see], [ 7]–[10]. Although most of the well-studied conjugative plasmids replicate in Gram-negative bacteria, an increasing interest in conjugative plasmids of Gram-positive bacteria has resulted in the recent analysis of conjugative plasmids from for instance streptococci, enterococci, staphylococci and clostridia [11]–[17]. However, conjugation systems present on the Gram-positive soil bacterium *Bacillus subtilis* had not been reported until recently. This is most remarkable taking into account that (i) it is one of the best-studied Gram-positive bacteria; (ii) it has important industrial applications; and (iii) it is closely related to pathogenic and fastidious bacilli [for review see], [ 18,19]. Moreover, several *B. subtilis* strains are gut commensals in animals including humans [20]. *B. subtilis* plasmids may therefore play an important role in HGT in different environments. We chose the *B. subtilis* plasmid pLS20 for our studies. Originally, this 65 kb plasmid was identified in the *Bacillus subtilis natto* strain IFO3335 that is used in the fermentation of soybeans to produce “natto”, a dish that is popular in South Asia [21]. Previous studies on pLS20 have shown that it is conjugative in liquid media as well as on solid media [22], [23]. The presence of pLS20 has a broad impact on the physiology of the host, and the localization of some components of the conjugation machinery has been determined [24], [25]. The replication region of pLS20 has been characterized, and it has been demonstrated that it uses a dedicated segregation mechanism involving the actin-like Alp7A protein [26], [27]. pLS20 encodes a protein, Rok_LS20_, that suppresses the development of natural competence of *B. subtilis* [28].

Recently, we have reported a global view of the regulatory circuitry of the pLS20 conjugation genes. A conjugation operon encompassing more than 40 genes is located next to a divergently oriented single gene, *rco_LS20_*, which encodes the master regulator of conjugation responsible for keeping conjugation in the default “OFF” state. Activation of conjugation requires an anti-repressor, Rap_LS20_, that belongs to the family of Rap proteins. Inactivation of the *rap_LS20_* gene on pLS20 severely compromises conjugation, and conjugation was enhanced when *rap_LS20_* was expressed from an ectopic locus. The activity of Rap_LS20_, in turn, is regulated by a signaling peptide, Phr*_LS20_. The small *phr_LS20_* gene, located immediately downstream of *rap_LS20_*, encodes a pre-protein. After being secreted, Phr_LS20_ can be processed by a second proteolytic cleavage, resulting in generation of the functional pentapeptide, Phr*_LS20_, corresponding to the five C-terminal residues of Phr_LS20_. When (re)imported, this peptide inactivates Rap_LS20_. Therefore, activation of conjugation is ultimately regulated by the Phr*_LS20_ signaling peptide. The Phr*_LS20_ concentration will be relatively high or low when donor cells are predominantly surrounded by donor or recipient cells, respectively. Hence, conjugation will become activated particularly under conditions in which recipient cells are potentially present. In addition, Phr*_LS20_ has a crucial role in returning conjugation to the default “OFF” state [29].

Despite identification of the players involved in regulation of the conjugation genes, our knowledge on regulation of the genetic switch responsible for activating conjugation is still very limited. Using a combination of various *in vitro* and *in vivo* approaches, we show that the genetic switch controlling pLS20 conjugation involves at least three layers of regulation. Together, they tightly repress the main conjugation promoter under conditions that do not favor conjugation, while maintaining the ability to accurately switch on the conjugation genes when appropriate conditions occur. The three layers involve coinciding or overlapping divergent promoters of different strengths, autoregulated expression of Rco_LS20_, which turns out to be a tri-functional transcriptional regulator, and formation of Rco_LS20_-mediated DNA looping. The sophisticated regulatory mechanism that combines three layers of control into a single switch is novel for plasmids of Gram-positive bacteria. The implications of the uncovered regulation mechanisms for conjugation are discussed in the context of regulatory systems present on other HGT elements and with other regulatory systems involving DNA looping.

## Results

### Promoters *P_c_* and *P_r_*

#### The *rco_LS20_*-gene *28* intergenic region contains the strong main conjugation promoter, *P_c_*, which is under the negative control of the master regulator of conjugation Rco_LS20_

**Figure 1:**
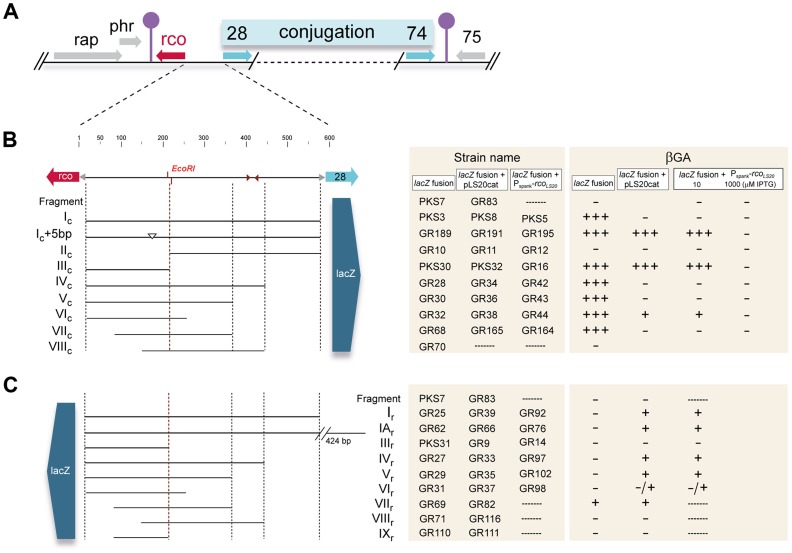
Genetic map of the pLS20cat conjugation region and summary of the transcriptional *lacZ* fusions used in this study. **A**. Map of the conjugation region of plasmid pLS20cat. Positions and directions of the genes and the positions of the predicted transcriptional terminators are indicated with arrows and lollipop symbols, respectively. Panels **B** and **C** show a blow-up of the 600 bp *rco_LS20_* gene *28* intergenic region and the different fragments fused to *lacZ*. Fusions in (**B**) and (**C**) were used to study activities of the promoters *P_c_* and *P_r_*, respectively. Features of the intergenic region are given on the top line. Numbers correspond to the bp position in this region. Names of the fragments cloned are indicated. The small triangles indicate position of an inverted repeated sequence (see text). Strains containing *P_c_*-*lacZ* fusions in combination with the *P_spank_*-*rco_LS20_* cassette were grown on plates containing 10 µM or 1 mM IPTG. The symbols “+++”, “+”,and “−” reflect intense blue, pale blue, and white colonies after growth on X-gal containing plates. Colors of the colonies were observed after 16 and 48 hours of incubation at 37°C for strains containing pLS20cat or the *P_spank_*-*rco_LS20_* cassette, respectively.

According to our standard presentation (Fig. 1A), the transcription of pLS20cat gene *27*, encoding the main repressor of conjugation genes, Rco_LS20_, reads leftwards. Flanking genes *28* to *74*, which are all transcribed in the opposite direction, probably constitute a large conjugation operon [29]. To test whether a promoter that would drive expression of this operon is located upstream of gene *28* we cloned the ∼600 bp intergenic *rco_LS20_*-gene *28* region in the appropriate orientation in front of a promoterless *lacZ* reporter, and subsequently placed a single copy of this cassette at the *B. subtilis* chromosomal *thrC* locus (strain PKS3). Transcriptional fusions to several sub-fragments of this region were also constructed. In addition, all the fragments were cloned in the opposite orientation to analyze the divergent promoter of the *rco_LS20_* gene (see below). For simplicity, the cloned fragments are indicated with Roman letters. Fragments cloned in the orientation to analyze the conjugation or the *rco_LS20_* promoter are indicated with the extension “c” or “r”, respectively. The entire intergenic region is referred to as Fragment I (or F_I). A schematic representation of the different strains and fusions described in this work is given in Figs. 1B–C.

Colonies of strain PKS3 containing F_I_c_-*lacZ* fusion were blue when grown overnight on Luria-Bertani (LB) agar plates supplemented with the chromogenic substrate 5-bromo-4-chloro-indolyl-β-D-galactopyranoside (Xgal; Fig. S1), demonstrating that the *rco_LS20_*-gene *28* intergenic region contains a promoter, which we named *P_c_*. Analysis of PKS3 samples gave relatively high levels of β-galactosidase (βG) activities that were in the range of 300 and 500 Miller Units (MU) during mid-exponential and stationary phase, respectively. These results indicate that *P_c_* is a rather strong promoter that does not seem to be regulated by host-encoded factors when grown under these conditions.

**Figure 2:**
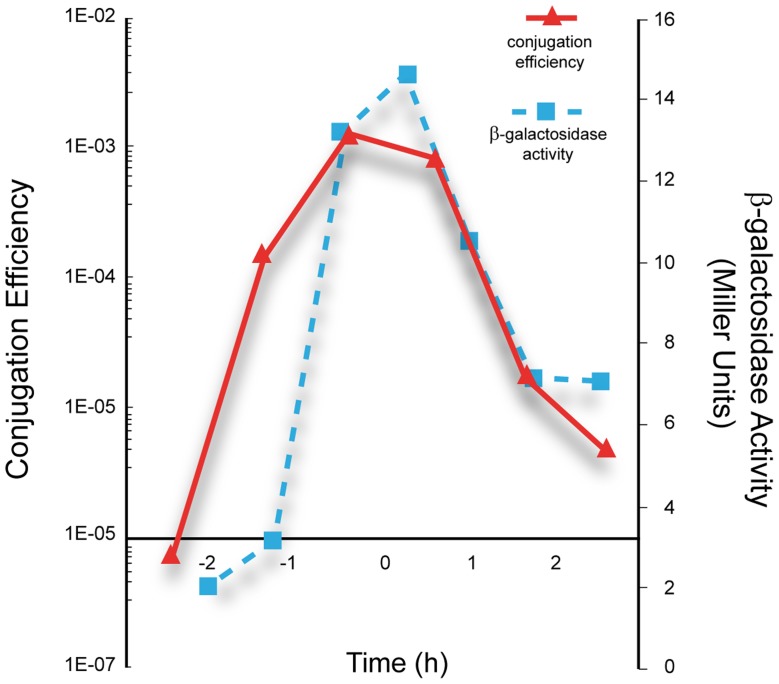
Correlation between the kinetics of *P_c_* promoter activity and conjugation efficiencies of pLS20cat. Overnight cultures of the strain PKS8 (F_I_c_-*lacZ*, pLS20cat) and recipient strain PS110 were diluted to an OD_600_ of 0.05. Next, samples taken at different times were used to determine conjugation efficiency of pLS20cat by a standard conjugation protocol (continuous line), and the promoter *P_c_* activity by measuring β-galactosidase activity (broken line). T = 0 corresponds to the end of the exponential growth phase. The presented graph corresponds to a representative experiment. The experiment was carried out three times and the corresponding values differed by less than 10%.

Under our laboratory conditions, efficient conjugation is limited to a narrow time window near the end of the exponential growth phase [29]. If *P_c_* is the main conjugation promoter it is expected that (i) its activity would generally be lower in the presence of pLS20cat and (ii) there would be a correlation between promoter *P_c_* activity and the efficiency of conjugation. The following results show that this is indeed the case. Thus, we introduced pLS20cat into strain PKS3, and colonies of the resulting strain, PKS8, were white after overnight growth on Xgal-containing plates (Fig. S1). In addition, when we used PKS8 as donor strain and simultaneously determined the kinetics of conjugation and promoter *P_c_* activity we found that promoter *P_c_* is only active during a rather short window of time near the end of the exponential growth phase, which coincides with the period of high conjugation efficiency (Fig. 2).

Next we tested whether Rco_LS20_, encoded by the pLS20cat gene *27* (*rco_LS20_*), is responsible for repression of the *P_c_* promoter. To this end, we placed the *rco_LS20_* gene under the control of the isopropyl β-D-1-thiogalactopyranoside (IPTG)-inducible *P_spank_* promoter and introduced this cassette at the *amyE* locus of strain PKS3 (harboring the F_I_c_-*lacZ* fusion at the *thrC* locus). Colonies of this strain, PKS5, were blue when grown on Xgal-supplemented LB agar plates, but white when the plates contained IPTG (Fig. S1). Together, these results indicate that promoter *P_c_* located upstream of gene *28* constitutes the main conjugation promoter that is negatively controlled by Rco_LS20_.

Interestingly, PKS5 colonies were white when plates contained as little as 10 µM of IPTG (colonies shown in Fig. S1). Taking also into account that *P_spank_* is a relatively weak promoter, these results indicate that the *P_c_* promoter is very sensitive to Rco_LS20_.

#### Promoter *P_c_* is located at an unusually large distance upstream of the first gene of the conjugation operon

We next set out to delineate the position of the *P_c_* promoter. As a first approach, we constructed strains containing *lacZ* fused to different subregions of Fragment I_c_. Surprisingly, whereas no significant promoter activity was obtained with the strain having *lacZ* fused to Fragment II_c_ (strain GR10), the βG activities obtained with strains harboring *lacZ* fused to Fragment III_c_, IV_c_, V_c_ or VI_c_ were very similar to those obtained when *lacZ* was fused to Fragment I_c_. These results show that promoter *P_c_* is located at an unusually large distance of at least 350 bp upstream of gene *28*.

**Figure 3:**
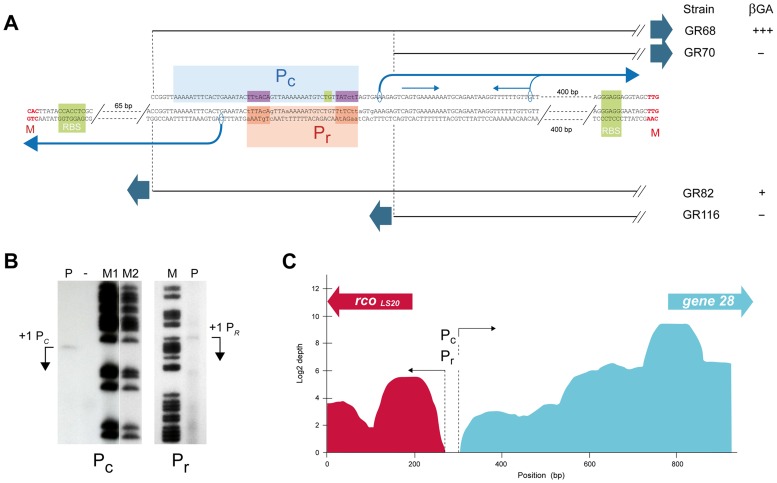
Promoter *P_c_* is located 461 bp upstream of the start codon of gene *28* and overlaps with the divergently oriented *P_r_* promoter. **A**. Determination of promoter *P_c_* and *P_r_* sequences by deletion analysis and primer extension. pLS20cat containing cells harvested at the end of the exponential growth phase were processed to isolate their total RNA, which was used in primer extension assays as described in Materials and Methods. Features of the promoter *P_c_* are shown above the sequence. The dotted vertical lines and black straight lines indicate the 5′ end points of the transcriptional *lacZ* fusions present in strains GR68 and GR70, displaying and not displaying promoter activity, respectively. The core promoter and the putative upstream UP element is indicated by a light blue box; the −35 and −10 hexamers, and the extended −10 motif are indicated with dark blue and green boxes, respectively. The transcription start site determined by primer extension and the direction of transcription are indicated with the corresponding encircled base and a black bent arrow. The thin grey bent arrow corresponds to the 3′ end point of the smaller extension product that coincides with the start of an inverted repeat which is marked with a pair of thin blue arrows above the sequence. Features of promoter *P_r_* are shown below the sequence. The dotted vertical lines and the black straight lines indicate the 3′ end points of the transcriptional fusions with *lacZ* reporter present in strains GR82 and GR116, displaying and not displaying promoter activity, respectively. The deduced position of the *P_r_* core promoter, and the −35 and −10 boxes are indicated with orange and red boxes, respectively (see text). The transcription start site determined by primer extension and the direction of transcription are indicated with the corresponding encircled base and a black bent arrow. **B**. Primer extension to determine the transcription start sites of promoters *P_c_* (left panel) and *P_r_* (right panel). The cDNA products of the primer extension reactions are indicated with bent arrows (lane P). Free lane in which no sample was run is indicated with “−”. Lanes M, M1 and M2 correspond to [G+A] chemical sequencing reactions of a short 230 bp DNA fragment corresponding to the studied pLS20cat region obtained by PCR amplification as described in Materials and Methods. In the case of the *P_c_* promoter, a smaller extension product with a relatively strong signal was observed 37 bp downstream of the extension product shown. The longer extension product most likely reflects the correct transcription start site based on the following arguments. First, it is known that AMV reverse transcriptase prematurely terminates cDNA synthesis when reaching a stem loop in the RNA, and that the prematurely terminated molecules map at the bottom of the secondary structure [71], [72]. The position of the strong signal coincides with the 3′ end of an inverted repeat (indicated in Fig. 3A). Second, no putative core promoter sequences are evident upstream of the 5′ position of the shorter extension product. Third, if the stronger signal corresponds to the transcription start site, the responsible promoter would be present on Fragment VIII_c_ used for the transcriptional *lacZ* fusion in strain GR70. However, no promoter activity was observed with this strain (see text). And fourth, the transcription start site based on the longer extension product corroborates the RNAseq data. **C**. Schematic overview of RNAseq expression data of pLS20cat genes *rco_LS20_* and *28* under conditions with (top panel) and without overexpression of *rco_LS20_* (lower panel). The amount of right and leftward “reads”, given in green and red, respectively, are presented on a log2 scale. The positions of the divergently oriented genes *rco_LS20_* and *28* are indicated on the top with a red and green arrow, respectively. Dotted lines and black arrows indicate the approximate start sites of the divergent transcripts driven by the *P_c_* and *P_r_* promoters.

Analyses of strains containing *lacZ* fused to Fragment VII_c_ (GR68) or VIII_c_ (GR70) revealed that promoter activity was sustained only by Fragment VIIc (Fig. 1B), showing that the 5′-located 63 bp region of Fragment VII_c_ contains (at least part of) the P_c_ promoter. This 63 bp region contains the sequence 5′- ttaaaaatttcactgaaatac-**TTtACA**-gttaaaaaaatgtc-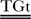 **TATctT**-3′, which constitutes a putative σ^A^-dependent promoter endorsing several features characteristic for a strong promoter. First, the hexamer sequences 5′-TTtACA-3′ and 5′-TATctT-3′ are very similar to the consensus −35 (5′-TTGACA-3′) and −10 (5′-TATAAT-3′) sequences recognized by σ^A^. Second, an optimal spacer length of 17 bp separates the putative −35 and −10 boxes. Third, the spacer contains the extended −10 motif (5′-TGn-3′, double underlined). Fourth, AT-rich tracts are located directly upstream of the predicted −35 box which are likely binding sites for the C-terminal domain of the RNA polymerase α-subunit. Additional evidence that this sequence constitutes the *P_c_* promoter was obtained by primer extension analysis to determine the transcription start site. The detected extension product is shown in Fig. 3B. The position of the deduced transcription start site is located 6 bp downstream of the *P_c_* core promoter sequences mentioned above (see Fig. 3A). The position of the transcription start site corroborates with our RNAseq data, which provides a good estimation of the position of the transcriptional start site. Thus, total RNA isolated from pLS20cat-harboring cells was processed as described in Materials and Methods after which it was employed to generate cDNA libraries using a “directional RNA-seq” procedure that preserves information about the transcript's direction. The schematic representation of the distribution and directionality of the reads presented in Figure 3C shows that the rightward-oriented transcripts, driving expression of gene *28* and downstream genes (shown in green), start close to the divergently oriented *rco_LS20_* gene (shown in red).

#### The *rco_LS20_*-*28* intergenic region contains the weak *P_r_* promoter that is activated and repressed at low or high Rco_LS20_ concentrations, respectively

As for *P_c_*, we constructed *lacZ* fusion strains to characterize the divergently oriented *P_r_* promoter responsible for expression of Rco_LS20_. Surprisingly, no promoter activity was observed when *lacZ* was fused to the 570 bp Fragment I_r_ (strain GR25, Fig. 1C). One possibility could be that promoter *P_r_* is located even further upstream. This does not seem to be the case however, because Fragment IA_r_, corresponding to the 1,014 bp region upstream of *rco_LS20_* (strain GR62), also did not provide detectable levels of promoter activity. We then introduced pLS20cat into these strains to study whether it encoded a protein that might be required to activate promoter *P_r_*. Colonies of the resulting pLS20cat-harboring strains GR39 (F_I_r_-*lacZ*) and GR66 (F_IA_r_-*lacZ*) turned pale blue when grown on Xgal-containing plates (Fig. 1C), consistent with pLS20cat providing a protein that activates the P_r_ promoter. In addition, the results show that the *P_r_* promoter is located on Fragment I_r_.

Rco_LS20_ might be responsible for activating its own promoter. To test this possibility, we engineered strain GR92 that contains the F_I_r_-*lac*Z fusion combined with the cassette in which expression of *rco_LS20_* is under the control of the IPTG-inducible *P_spank_* promoter. Colonies of strain GR92 were white when grown on agar plates containing only Xgal, but turned pale blue when the plates contained also low levels of IPTG. These results demonstrate that Rco_LS20_ activates its own promoter. In addition, the fact that colonies only developed a pale blue color suggests that promoter *P_r_* is weaker than *P_c_*. To test this more directly, we measured *P_r_* promoter activities at late-exponential growth phase using strain GR92 grown at different levels of Rco_LS20_ induction (Table 1). Interestingly, maximum *P_r_* promoter activity was obtained when cells were grown in the presence of 50 µM IPTG. Promoter *P_r_* activity decreased at higher IPTG concentrations and equaled background levels in the presence of 1 mM of IPTG, indicating that Rco_LS20_ represses its own promoter at higher concentrations.

**Table 1:** *P_r_* promoter activity at different induction levels of Rco_LS20_.

[IPTG] (µM)	βGA (MU)
0	<0.1
10	0.2
20	0.7
50	1.2
100	0.4
200	0.3
500	0.2
1.000	<0.1

Overnight culture of GR92 cells grown in the absence of IPTG was diluted 100-fold in fresh prewarmed LB medium containing the indicated amount of IPTG at 37°C. βGA was determined for samples withdrawn at late exponential growth (0D600 = ∼0.8). Background levels obtained with negative control strain PKS7 were <0.1 MU. Values are the mean of three independent experiments and for each point fluctuations were less than 10%. βGA, β galactosidase activity.

Together, these results show that *P_r_* is a weak promoter whose strength is several hundred folds weaker than that of *P_c_*. The results also show that Rco_LS20_ has a triple function. First, low levels of Rco_LS20_ are required to activate its own promoter *P_r_*; second, at higher concentrations Rco_LS20_ represses its own promoter; and third, Rco_LS20_ is responsible for repression of the oppositely oriented *P_c_* promoter. This triple function of Rco_LS20_ is likely to have important consequences for regulation of the conjugation genes (see discussion). It is worth mentioning that whereas maximum activation of the *P_r_* promoter was achieved when *rco_LS20_* was induced from the *P_spank_* promoter at 50 µM IPTG, efficient repression of the *P_c_* promoter was observed by inducing *rco_LS20_* with as low as 10 µM IPTG. Finally, the results obtained show that Rco_LS20_ is the only pLS20cat protein required for activation and repression of the *P_r_* and *P_c_* promoters.

#### The divergent *P_r_* and *P_c_* promoters overlap

As a first approach to determine the position of the *P_r_* promoter we constructed strains containing *lacZ* gene preceded by different subregions of Fragment I_r_ combined with pLS20cat to provide Rco_LS20_ in *trans*. The transcriptional regulator Rco_LS20_ is a DNA binding protein (see below). Therefore, a lack of *P_r_* promoter activity in the reporter assay can be due to the absence of (part of) the *P_r_* promoter or the Rco_LS20_ binding sites required for activation of *P_r_*. Since activator proteins generally bind upstream of promoters, we tested constructs having deletions at the 3′ end of Fragment I_r_ (i.e. flanking the *rco_LS20_* gene). Promoter *P_r_* activity was detected when *lacZ* was fused to Fragment VII_r_ (strain GR82), but not when it was fused to Fragment VIII_r_ (strain GR116) (Figs. 1C and 3A). Interestingly, these results suggested that promoter *P_r_* would be (partially) located on the 63 bp 5′ region of Fragment VII on which the divergently oriented *P_c_* promoter is also located (see above, Fig. 3A). In a complementary approach, we determined the transcriptional start site of promoter *P_r_* by primer extension (Fig. 3B). The determined transcription start site of promoter *P_r_* is positioned 6 bp upstream of the −35 box of the *P_c_* promoter (see Fig. 3A). This implies that promoter *P_r_* overlaps with the *P_c_* promoter.

*P_r_* is a weak promoter whose activity requires Rco_LS20_ (see above). It is therefore unlikely that the −35 and −10 boxes will be very similar to the consensus sequences. The following two sequences that may constitute a σ^A^-dependent promoter are located upstream of the determined *P_r_* transcription start site: (i) [5′-aaGAtA- 17 bp -TgTAAa-3′] and (ii) [5′-aTaACA-18bp-aAgtAT-3′] (mismatches with respect to consensus −35 (5′-TTGACA-3′) and −10 boxes (5′-TATAAT-3′) given in lower case, see Fig. 3A). The position of the determined transcription start is optimally spaced with respect to the first but not the second possible promoter sequence. Therefore, we favor the first sequence to correspond to the *P_r_* promoter. Interestingly, this would imply that the positions of the −10 and −35 boxes correspond exactly to the −35 and −10 boxes, respectively, of the divergently oriented *P_c_* promoter.

The results of the RNAseq experiments presented in Figure 3C (see also above) support the conclusion that the *P_r_* and *P_c_* promoters overlap. RNA transcripts mapped against the entire intergenic region except for a small region that is located near the start of the *rco_LS20_* gene. The divergent promoters *P_r_* and *P_c_*, responsible for the left- (red) and rightward (green) oriented transcripts, respectively, must both be located in the small nontranscribed region which corresponds to the position of the *P_r_*/*P_c_* promoters according to their transcriptional start sites determined by primer extension.

In summary, results obtained by a combination of different approaches demonstrate that divergent *P_c_* and *P_r_* promoters overlap, if not coincide.

### Rco_LS20_ operator sites

#### *In vivo* evidence that Rco_LS20_ binds to two operator sites; one of them, -located more than 85 bp downstream of *P_c_*-, is required for efficient regulation of promoters *P_c_* and *P_r_*

Rco_LS20_ belongs to the Xre-family of transcriptional regulators and is predicted to contain a Helix-Turn-Helix (HTH) DNA binding motif in its N-terminal region [29]. It is therefore likely that Rco_LS20_ will exert its transcriptional regulatory effects on *P_r_* and *P_c_* by binding to DNA sequences. We employed the following *in vivo* approach to gain insights into the location of the Rco_LS20_ binding sites. Either pLS20cat or the *P_spank_*-*rco_LS20_* cassette was introduced into the various *lacZ* fusion strains (see Fig. 1). The resulting strains were then grown on Xgal containing LB plates, -supplemented with or without 10 µM of IPTG for strains containing the *P_spank_*-*rco_LS20_* cassette-, and expression of the different *lacZ* fusions in response to Rco_LS20_ was screened by the color of their colonies.

A schematic summary of the results obtained for promoter *P_c_* is given in Figure 1B. In agreement with results presented above, the strain harboring *lacZ* fused to Fragment I_c_ (PKS3) displayed high *P_c_* promoter activity, but no promoter activity was detected when Rco_LS20_ was provided in *trans* (strains PKS5 and PKS8). Efficient Rco_LS20_-mediated repression of the *P_c_* promoter was lost however when *lacZ* was fused to Fragment III_c_ (strains GR16 and PKS32). This strongly indicates that an Rco_LS20_ operator site is located on the 368 bp Fragment II_c_ and that this operator, which would be located at least 85 bp downstream of the *P_c_* promoter, is crucial for efficient repression of the *P_c_* promoter. Fragment II_c_ contains an inverted repeated sequence (5′-ATCAAAATCAtgctgcaactTGGTTTTGAT-3′). To test whether this region constitutes an Rco_LS20_ operator site we constructed *lacZ* fusions to Fragments IV_c_ or V_c_, and also engineered derivatives of these two strains containing pLS20cat or *P_spank_*-*rco_LS20_*. The 5′ ends of these Fragments are located up- or downstream of the inverted repeat (see Fig. 1B). The *P_c_* promoter present on Fragment IV_c_ and V_c_ was efficiently repressed by Rco_LS20_, indicating that the Rco_LS20_ operator site is present on Fragment V and not on the 212 bp region immediately upstream of gene *28* containing the mentioned inverted repeat. Efficient Rco_LS20_-mediated repression of promoter *P_c_* was not observed for the *lacZ* fusion based on Fragment III_c_ (see above). Together these *in vivo* results strongly indicate that an ∼160 bp region, located 85 bp downstream of *P_c_*, contains an Rco_LS20_ operator site that is required for efficient repression of this promoter. We name this operator site O_I_.

Results described above show that promoter *P_c_* was not repressed by Rco_LS20_ when the *lacZ* fusion was based on Fragment III_c_ (strain GR16) and cells were grown in the presence of 10 µM IPTG. Interestingly though, promoter *P_c_* in strain GR16 was efficiently repressed when the concentration of IPTG was increased to 1 mM (see Fig. 1B). This indicates that another Rco_LS20_ operator site is present on the 201 bp Fragment III_c_. We name this operator site O_II_.

Next, we used the same strategy to delineate the regions required for activation of the divergent *P_r_* promoter. The results of these analyses are summarized in Figure 1C. Interestingly, the region required for efficient repression of *P_c_* by Rco_LS20_, is also required for Rco_LS20_-mediated activation of promoter *P_r_*. Thus, Rco_LS20_ activated the *P_r_* promoter when *lacZ* was fused to Fragments IV_r_ or V_r_ (strains GR97/GR33 and GR102/GR35, respectively) but not when it was fused to Fragment III_r_ (strains GR14/GR9, Fig. 1C). In summary, the *in vivo* results obtained provide strong evidence that one Rco_LS20_ operator, O_I_, is located in an ∼160 bp region located 85 bp downstream of promoter *P_c_*, and that this operator is crucial for proper repression and activation of promoters *P_c_* and *P_r_*, respectively. In addition, the results indicate the presence of another Rco_LS20_ operator, O_II_, which would be located near promoters *P_c_* and *P_r_*.

#### *In vitro* approaches show that Rco_LS20_ binds cooperatively to multiple binding sites present in operators O_I_ and O_II_

To study the position of the Rco_LS20_ binding sites in more detail we purified Rco_LS20_ and used it in Electrophoretic Mobility Shift Assays (EMSA). To facilitate purification, we constructed an *E. coli* strain that expresses an Rco_LS20_-His_(6)_ tagged fusion protein. The *his_(6)_*-tag was placed at the C-terminus because Rco_LS20_ contains a predicted Helix-Turn-Helix DNA binding motif close to its N-terminus. The following result demonstrates that the Rco_LS20_-His_(6)_ protein is functional *in vivo*. We constructed *B. subtilis* strain GR90 in which the expression of *rco_LS20_-his_(6)_* gene is placed under the control of the inducible *P_spank_* promoter, and which also contains the F_I_c_-*lacZ* reporter fusion. The activity of promoter *P_c_* in strain GR90 was repressed in an IPTG-dependent manner similar to that observed for strain PKS5 containing an inducible copy of native *rco_LS20_* (not shown).

**Figure 4:**
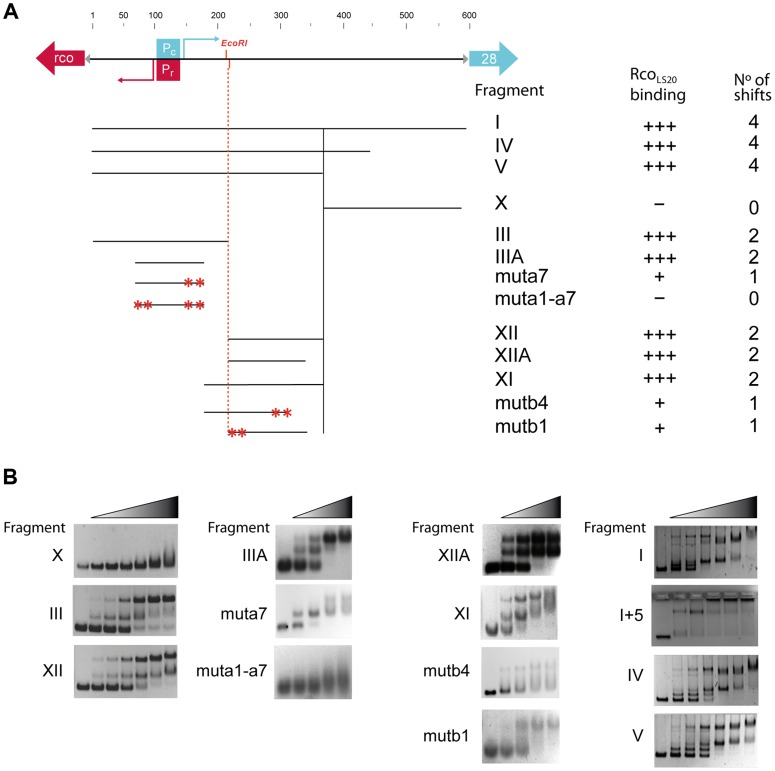
Analysis of the Rco_LS20_ binding sites in the *rco_LS20_* - gene *28* intergenic region by EMSA. **A**. Schematic representation of the DNA fragments used as probes and a summary of the EMSA results. Top panel. A map of the 600 bp *rco_LS20_*-gene *28* intergenic region is shown as a black line alongside with the positions of the characteristics in this region. The divergently oriented genes *rco_LS20_* and *28* are indicated with a red and blue arrow, respectively. The triangles indicate ribosomal binding sites. The position of the *P_c_* and the *P_r_* promoter (boxed) and their transcription start sites (bent arrows) are indicated in blue and red, respectively. The position of the unique *Eco*RI site in this region is also indicated. Lower panel shows the different fragments used in EMSA; the names of the DNA fragments, the levels of Rco_LS20_ binding and the numbers of shifts are indicated on the right. Asterisks indicate positions of introduced mutations. **B**. Examples of the EMSA results obtained using DNA fragments described in panel A. The left-most lane of each panel corresponds to samples loaded without protein. Increasing concentrations of Rco_LS20_ were prepared using a two-fold dilution method, and ranged from 0.212–6.8 (7 lane panels) or 0.95–7.3 µM (5 lane panels).

The *in vivo* transcriptional fusion results presented above indicated the presence of two operators. One of them, operator O_II_, located near promoters *P_c_*/*P_r_*, and another one, operator O_I_, present in an ∼160 bp region about 85 bp downstream of *P_c_*. In addition, this analysis indicated that the ∼200 bp region immediately upstream of gene *28* does not contain Rco_LS20_ binding sites. Accordingly, we began analyzing binding of Rco_LS20_ to Fragments X (200 bp region upstream gene *28*), III (expected to contain operator O_II_) and XII (expected to contain operator O_I_) (see Fig. 4). Independent of the concentrations used, Rco_LS20_ did not bind to Fragment X (Fig. 4B). Together with the *in vivo* data presented above, this provides strong evidence that this region does not contain Rco_LS20_ binding sites. Also in agreement with the *in vivo* data, Rco_LS20_ bound to both Fragment III and Fragment XII (Fig. 4B). Interestingly, the retardation patterns obtained for these fragments were similar, and resulted in the appearance of a maximum of two retarded species. The observation that the two retarded species were already present at low Rco_LS20_ concentrations when the majority of the DNA migrated to the position of unbound DNA, strongly indicates that Rco_LS20_ binds cooperatively to at least two binding sites present in each operator. In addition, the observation that DNA fragments entered the gel even at very high protein concentrations indicates that Rco_LS20_ binds to specific sites and that it does not spread along the DNA. To delineate the O_I_ and O_II_ regions further we used overlapping and subregions of Fragments III and XII as probes. Fragment IIIA (130 bp containing promoters P_c_/P_r_) and Fragment XIIA (125 bp) both produced up to two shifts, and Rco_LS20_ did not bind to the 46 bp region that separates these two fragments. This latter conclusion is based on comparison of gel retardations obtained with fragments XI and XII.

We next analyzed binding of Rco_LS20_ to Fragments I, IV and V that encompass both operators. These fragments gave similar retardation patterns. Interestingly, in these cases, Rco_LS20_ binding resulted in the appearance of four retarded species. All four of these retarded species could be detected at low Rco_LS20_ concentrations when most of the fragment had not bound Rco_LS20_, indicating that Rco_LS20_ binds cooperatively to multiple sites on these fragments.

**Figure 5:**
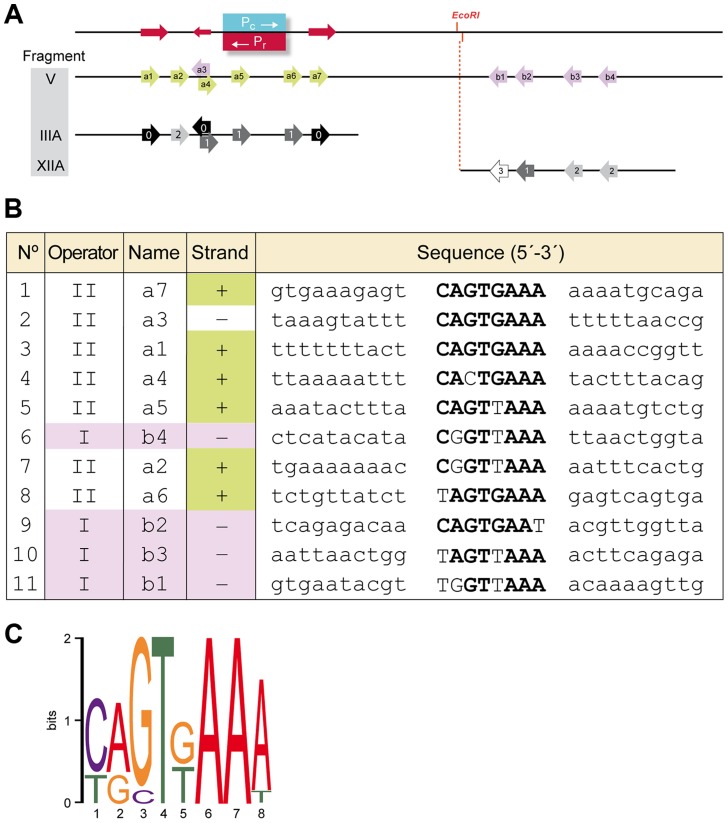
Identification of a conserved motif constituting (part of) the Rco_LS20_ binding site. **A**. Schematic representation of the region corresponding to Fragment V, which encompasses promoters *P_r_*/*P_c_* and the identified repeated motif that form (part of) the binding site for Rco_LS20_. Top line. Position of promoters *P_c_* and *P_r_* are indicated in blue and red, respectively. The position of the unique *Eco*RI site is indicated. A 13 bp long direct repeat (5′-TCAGTGAAAAAAA-3′) is indicated with rightward directed red arrows. The leftward-directed arrow indicates the position of the complementary 9 bp sequence 5′-TTTCACTGA-3′. Second line (Fragment V). Arrows indicate the positions of the identified motifs a1–a7 and b1–b4. Motifs present on the upper and the lower strand are shown in green and purple, respectively. Third and fourth line show identified motifs present on Fragment III_A and XII_A, respectively. Black, dark grey, grey and light grey indicate motifs identical to the consensus sequence or deviating at one, two or three positions, respectively. **B**. An alignment of the nucleotide sequences of the eleven identified motifs and their flanking sequences. Names according to the nomenclature in “A” are given together with information on the strand and the region. Sequences corresponding to the consensus sequence of the motif are given in bold. **C**. A representation of the consensus motif generated by Weblogo [73]. The size of each nucleotide corresponds to the frequency with which that nucleotide is observed in that position.

To search for the presence of conserved motifs in the two Rco_LS20_ operators we used the motif-identification programs MEME [30] and BIOPROSPECTOR [31]. These analyses revealed the identification of an 8 bp conserved motif that is present seven times in O_II_ (Fragment III_A), and four times in O_I_ (Fragment XII_A). We named the seven motifs identified in the O_II_ operator a1–a7, and the four motifs in the O_I_ operator b1–b4 (see Fig. 5). Whereas motifs b1 to b4 are all located on the lower strand, motifs a1–a7 are located on the upper strand, except motif a3. It is worth mentioning some characteristics of motifs a1 to a7. First, motif a5 overlaps with the *P_c_*/*P_r_* core promoter sequences, and motifs a1–a4 and a6–a7 flank them. Second, motifs a1 and a7 form part of a 13 bp direct repeat. Third, motifs a1 and a3 form an inverted repeat. Fourth, the oppositely oriented motifs a3 and a4 overlap in a region that has an inverted repeat (5′TTTCAgTGAAA-3′).

Evidence that the identified motif constitutes (part of) the binding site for Rco_LS20_ was obtained by DNase I footprinting (see below) and mutational analysis. Thus, gel retardation assays showed that binding of Rco_LS20_ is affected in probes containing alterations in one or two motifs in either operator. For instance, Rco_LS20_ did not bind to a derivative of Fragment III_A containing mutations in both motifs a1 and a7; and binding was affected when only motif a7 was mutated. Similarly, mutation of motif b1 or b4 resulted in the appearance of only one retarded species instead of two observed for corresponding fragments without mutations (Fig. 4B). In summary, the results obtained show that the intergenic *rco_LS20_*-gene *28* region contains two Rco_LS20_ operators that are separated by 75 bp. Operator O_II_ overlaps with promoters *P_r_*/*P_c_* and the other region is located 75 bp towards the direction of gene *28*. Each region contains repeats of a motif whose consensus sequence is 5′-CAGTGAAA-3′ and which forms (part of) the binding site of Rco_LS20_. Motifs in O_I_ are located on the lower strand, and except for one, motifs in O_II_ are located on the upper strand.

**Figure 6:**
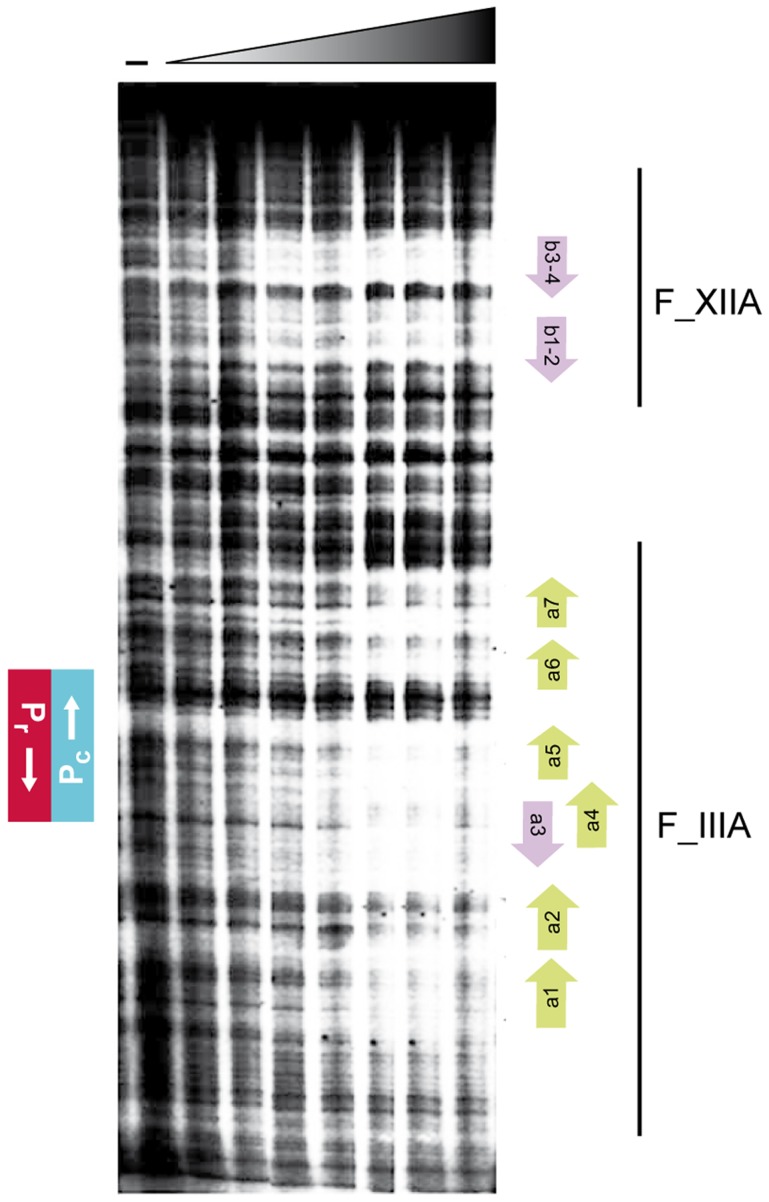
Footprint analyses of the binding of Rco_LS20_ to the *rco_LS20_* - -gene *28* intergenic region. Fragment V, end-labeled at the *P_c_* template strand, was analyzed for binding of Rco_LS20_-His. First lane (−) was not incubated with protein. Concentrations of Rco_LS20_-His, increasing by two-folds, ranged from 0.11 to 7.04 µM. The positions of the *P_c_* and *P_r_* promoters are indicated on the left. Bars on the right reflect the regions covered by Fragments IIIA (F_IIIA) and XIIA (F_XIIA). Positions of motifs a1–a7 and b1–b4 are indicated with green or purple arrows at the right.

Binding of Rco_LS20_ to operators O_I_ and O_II_ was confirmed by DNase I footprinting. The results presented in Figure 6 confirm that Rco_LS20_ binds to a region that overlaps with the *P_r_*/*P_c_* promoters and to another region located about 75 bp downstream of the *P_c_* promoter. The combined *in vitro* results are in line with the *in vivo* results presented above.

#### Evidences that proper regulation of the *P_r_*/*P_c_* promoters involves DNA looping

**Figure 7:**
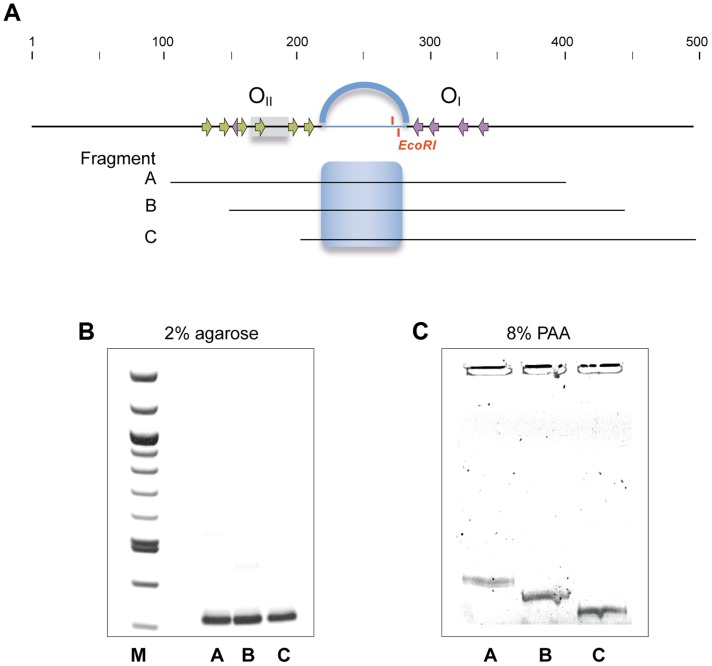
The 75 bp spacer region separating operators O_I_ and O_II_ contains a static bent. **A**. Schematic representation of the region encompassing operators O_I_ and O_II_ and the regions corresponding to the DNA fragments amplified by PCR that were subjected to 2% agarose (**B**) and 8% native PAA (**C**). Position of the *P_c_*/*P_r_* promoters and the Rco_LS20_ binding motifs within the operators are indicated with grey rectangles and arrows, respectively. The 75 bp region separating O_I_ and O_II_ is shown as an interrupted line and the position of the unique *Eco*RI site is given. The predicted curvature in this region is represented by the blue arc above the top line, and by a blue shading in the equivalent region in fragments A–C. Fragments A–C were run on 2% agarose or on 8% native PAA gel followed by ethidium bromide staining.

Operator O_I_, -located at a distance of more than 75 bp from *P_r_*/*P_c_*-, is needed for proper regulation of these promoters. This and other data presented above, suggest that proper regulation of the *P_r_*/*P_c_* promoters involves DNA looping mediated by Rco_LS20_ bound to operators O_I_ and O_II_. Due to the intrinsic stiffness of DNA, loops are generally longer than 90 bp because the curvature energy required to make smaller loops is too large, unless the DNA region separating the two operator sites is bent [32]. Operators O_I_ and O_II_ are separated by only 75 bp. Several periodically spaced A/T tracts can result in formation of a static bent [33]. The spacer region contains periodically spaced A/T tracts, and computer-assisted analysis predicts that the spacer region forms a static curve (see Fig. S2). These data prompted us to perform circular permutation assays. Thus, three overlapping fragments of identical size (314 bp) were generated in which the predicted static curve is located at different positions (see Fig. 7A). As expected, these fragments migrated to identical positions when run on a 2% agarose gel (Fig. 7B). However, when run on a native 8% PAA gel the fragments migrated differently and all of them run slower than expected for their size, with the fragment containing the predicted bent in the middle of the fragment migrating slowest (Fig. 7C). These results show that the 75 bp spacer contains a static bent.

**Figure 8:**
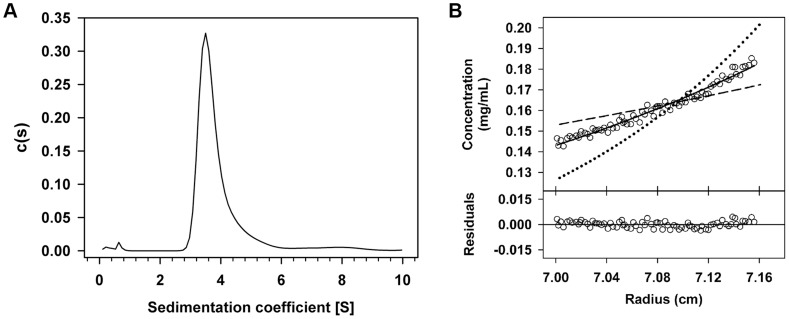
Rco_LS20_ forms tetramers in solution. The oligomerization state of Rco_LS20_ protein in solution was studied by two complementary analytical ultracentrifugation assays. **A**. Sedimentation velocity assay. Sedimentation coefficient distribution c(s) profile corresponding to 10 µM purified Rco_LS20_. **B**. Sedimentation equilibrium assay. Upper part: Sedimentation equilibrium data for Rco_LS20_ (empty circles) are presented together with best-fit analysis assuming protein dimer (dashed line), tetramer (black line), or octamer (dotted line) species. The data indicate that Rco_LS20_ is a tetramer at 10 µM. Lower part: The difference between estimated values and experimental data for protein tetramers (residuals).

If Rco_LS20_-mediated DNA looping occurs then it is expected (i) that Rco_LS20_ will form oligomers thereby creating a DNA binding unit able to bind simultaneously to O_I_ and O_II_, and (ii) that the two operators are in phase such that the Rco_LS20_ binding sites have a spatial orientation that is optimal for Rco_LS20_ binding. We tested both predictions. The oligomerization state of Rco_LS20_ was studied by two complementary analytical ultracentrifugation approaches (Fig. 8). In sedimentation velocity experiments, Rco_LS20_ was observed as a single species with an experimental sedimentation coefficient of 3.8 S. This value corrected to standard conditions (*s*_20,*w*_ = 4.1S) was compatible with an elongated protein tetramer (Fig. 8A). To confirm this result, sedimentation equilibration experiments were carried out within the concentration range from 10 to 30 µM. The calculated average molecular mass obtained was 85,200 Da±1,700, which corresponds to the tetrameric form of Rco_LS20_ (Fig. 8B).

To test if a specific phasing between O_I_ and O_II_ is important for Rco_LS20_ to carry out its regulatory role we constructed a derivative of Fragment I, I+5, in which we enlarged the spacer half a helical turn by inserting 5 bp and cloned this fragment in front of *lacZ* (see Fig. 1B). Next, we tested the responsiveness of promoter *P_c_* to Rco_LS20_ using strains containing either Fragment F_I_c_ or F_I_c_ +5 fused to *lacZ*. As expected, Rco_LS20_, which was provided *in trans* by pLS20cat, efficiently repressed promoter *P_c_* when *lacZ* was fused to Fragment I_c_ (strain PKS8). Promoter *P_c_* was not efficiently repressed by Rco_LS20_ however, when *lacZ* was fused to Fragment I_c_+5 (strain GR191). Thus, colonies of pLS20cat-harboring cells were blue when grown on Xgal-containing plates (see Fig. S3). These results show that enlarging the distance between O_I_ and O_II_ with half a helical turn destroys proper regulation of promoter *P_c_* by Rco_LS20_. Besides affecting the phasing, the 5 bp insertion might also affect the static curvature of the spacer region. Regardless whether the loss of Rco_LS20_-mediated regulation is due to phasing and/or altered curvature, the results provide compelling evidence that Rco_LS20_ mediates its regulatory effect through DNA looping.

Next, we analyzed by EMSA if the 5 bp insertion between operators O_I_ and O_II_ affects Rco_LS20_ binding. As described above, even in the presence of the highest Rco_LS20_ concentration applied, DNA fragments F_I, F_IV and F_V containing operators O_I_ and O_II_ entered the gel migrating to distinct positions, indicating multiple intramolecular Rco_LS20_ binding events (Fig. 4B, right column, first, third and fourth panel). Interestingly, however, whereas Fragment I+5 entered the gel at low Rco_LS20_ concentrations, most of the DNA did not enter the gel at medium or high Rco_LS20_ concentrations (Fig. 4B, right column, second panel). One possible explanation is that dephasing between the two operators allows Rco_LS20_ to bind intermolecularly resulting in the formation of high molecular weight nucleoprotein complexes that do not enter the gel. Together, these results support the view that the phasing between O_I_ and O_II_ is crucial for proper Rco_LS20_-mediated regulation of transcription.

## Discussion

**Figure 9:**
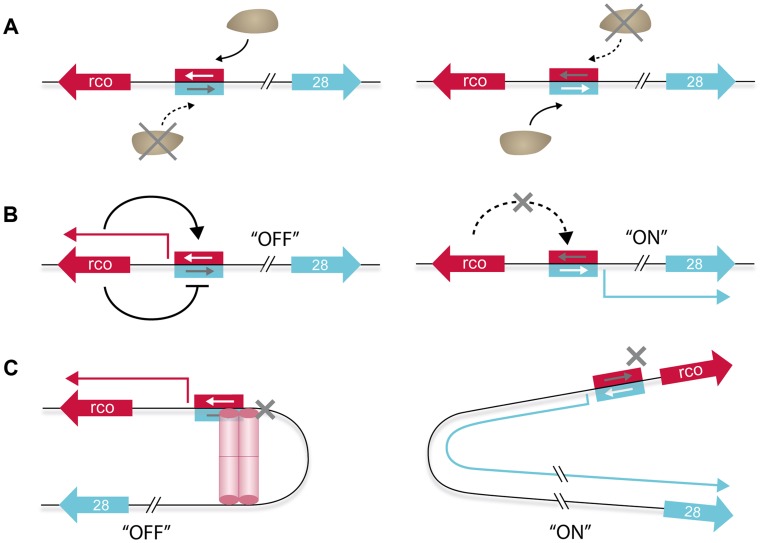
Model of the different layers contributing to the genetic switch controlling expression of the pLS20 conjugation genes. **A**. RNA polymerase acts itself as a switch because it is unable to bind simultaneously to both of the two overlapping and divergently oriented promoters. Consequently, RNA polymerase (the brown ellipse shaped form) binds only one promoter at a time resulting in transcription of only the gene(s) controlled by this promoter. **B**. Rco_LS20_ generates a self-sustaining positive feedback loop by activating transcription from its own promoter (*P_r_*) (left panel). This, combined with the simultaneous repression of the divergent conjugation promoter (*P_c_*), results in conjugation being maintained effectively in the “OFF” state. Relief of Rco_LS20_-mediated repression of the *P_c_* promoter results in activation of the conjugation genes (right panel). In addition, this interrupts the auto-stimulation of the *P_r_* promoter, preventing further synthesis of Rco_LS20_, which in turn will contribute in pushing and maintaining conjugation in the “ON” state. The negative auto-regulatory loop of Rco_LS20_ that probably functions to keep Rco_LS20_ within a low concentration range (see text) is not presented. **C**. DNA looping results in a high local concentration of Rco_LS20_, increasing specificity and affinity that dampens transcriptional fluctuations between and within individual cells (left panel). This would contribute to tight repression of the *P_c_* promoter, keeping conjugation in the “OFF” state under conditions antithetic to conjugation without compromising the ability to switching rapidly to a high expression state (i.e. “ON”, right panel) of the conjugation genes when appropriate conditions occur. *rco_LS20_* and gene *28*, -the first gene of the conjugation operon-, are indicated with large red and blue arrows, respectively. The same coloring scheme is used for the corresponding promoters (rectangular) and transcripts (thin broken arrows). Activation and repression of transcription are indicated with continuous black lines ending in an arrow and a “T” shape, respectively. The red cylindrical structures, which may reflect one or two Rco_LS20_ tetramers, represent the Rco_LS20_ oligomer mediating DNA looping.

Conjugation is a complex and energy consuming process, involving the generation and transfer of ssDNA, synthesis and assembly of a sophisticated type IV secretion system, and establishment of specific contacts with the recipient cell. Hence, the process of conjugation and expression of the genes involved are strictly controlled. Analysis of the regulation of conjugation genes present on ICEs in bacteria and those on plasmids of Gram-negative bacteria indeed indicates that this is the case [for review see], [ 5,7]. In our previous studies, we have sequenced and annotated plasmid pLS20cat of the Gram-positive bacterium *B. subtilis* and identified a large conjugation operon. We have also identified *rco_LS20_* as the gene encoding the master regulator of conjugation, Rap_LS20_ as the anti-repressor required to activate the conjugation genes, and we showed that the activity of Rap_LS20_ is in turn regulated by the signaling peptide Phr*_LS20_. In this study, we analyzed the underlying molecular mechanism of how the pLS20 conjugation genes are regulated. The results obtained provide compelling evidence that the conjugation genes of pLS20 are controlled by a complex genetic switch, which is composed of at least three intertwined layers. A scheme of the three layers is shown in Figure 9. One of the levels results from the relative positioning of the main conjugation promoter, *P_c_*, and the divergently oriented promoter *P_r_*, driving expression of the *rco_LS20_* gene (Fig. 9A). The presence of divergently oriented promoters is a common form of gene organization in bacteria, and the (likely) role of this organization in transcriptional regulation has long been recognized [34]. Nevertheless, direct proof for and detailed analysis of the implications on transcriptional regulation are restricted to only a minor fraction of the divergently oriented transcriptional units detected. Here, we identified the conjugation promoter *P_c_* and showed that it is a relatively strong promoter, which is repressed by the master regulator of conjugation Rco_LS20_. Importantly, the position of promoter *P_c_* coincides, or at least partially overlaps, with the divergently oriented weak *P_r_* promoter. It has been demonstrated that an RNA polymerase can bind only to one of two overlapping promoters [35], [36]. Thus, in the special configuration of overlapping promoters the RNA polymerase may itself act as a transcriptional regulator. Recently, Bendtsen *et al.* [37] described theoretical scenarios backed up by experimental data that overlapping promoters indeed can result in a transcriptional switch, provided that they have different activities in the absence of the regulatory protein, combined with a regulator that has a strong differential effect on the regulation of both promoters. This is exactly the case for the *P_c_*/*P_r_* promoter pair; in the absence of the regulator promoter *P_c_* is several hundred folds stronger than P_r_, and the presence of the regulator strongly represses the *P_c_* promoter while activating the *P_r_* promoter.

The second level of regulation contributing to the genetic switch concerns the multiple roles that Rco_LS20_ plays in the *P_c_*/*P_r_* regulation (Fig. 9B). We showed that, on the one hand, Rco_LS20_ activates transcription of its own weak promoter, *P_r_*, thereby generating a self-sustaining positive feedback loop. On the other hand, Rco_LS20_ functions simultaneously as an efficient repressor of the *P_c_* promoter. The dual effect that Rco_LS20_ has on *P_c_* and *P_r_* maintains conjugation effectively in the “OFF” state. We also showed that the level of *rco_LS20_* induction from an inducible promoter required for efficient repression of the *P_c_* promoter was about ten-fold lower than that required for maximum auto-activation of the *P_r_* promoter. These differential effects of Rco_LS20_ on repressing and activating the *P_c_*/*P_r_* promoters will also contribute towards maintaining conjugation stably in the “OFF” state under conditions when conjugation should not be activated. Interestingly, we found that at elevated concentrations Rco_LS0_ inhibits its own transcription. This negative autoregulation probably functions to keep Rco_LS20_ within a low concentration range in order to respond accurately to the anti-repressor Rap_LS20_ to activate the conjugation genes. The triple effects Rco_LS20_ has on the regulation of the *P_c_*/*P_r_* promoters will also play an important role when Rap_LS20_ induces the system to switch to the “ON” state. In addition to relieving repression of the strong conjugation *P_c_* promoter, this will simultaneously annihilate autostimulation of the *P_r_* promoter, preventing further synthesis of Rco_LS20_, which in turn will contribute in pushing and maintaining conjugation in the “ON” state.

A third level contributing to the genetic switch to activate the conjugation genes involves the DNA looping mediated by simultaneous binding of Rco_LS20_ to operators O_I_ and O_II_ (Fig. 9C). DNA looping mediated by a transcriptional regulator has been reported for several other regulatory systems in prokaryotes and their analyses have revealed that several features are conserved and necessary for DNA looping to occur [for review see, 38]. Our results showed that the properties of Rco_LS20_ and the DNA in the *P_c_*/*P_r_* region comply with the necessary features for Rco_LS20_-mediated loop formation. First, using different techniques, we show that Rco_LS20_, -predicted to contain a helix-turn-helix DNA binding motif in its N-terminal region [29]-, is a DNA binding protein and that it binds specifically to two operators, O_I_ and O_II_. Second, operator O_I_, which is located more than 85 bp away from promoters *P_c_* and *P_r_*, is required for efficient regulation of both promoters. Third, Rco_LS20_ binds cooperatively to both operators. Fourth, dephasing the positions of the two operators by inserting 5 bp in the spacer region destroys proper regulation of the conjugation genes. And fifth, we showed that Rco_LS20_ forms tetramers in solution. This will create a unit containing multiple DNA binding motifs, facilitating cooperative binding to multiple sites within the two operators.

The DNA loop in the *P_c_*/*P_r_* region of pLS20 is characterized by a small spacer region that separates Rco_LS20_ operators O_I_ and O_II_. The spacer length can be used to classify DNA loops into two categories: short or energetic loops, and long or entropic ones. Due to intrinsic stiffness and torsional rigidity of the DNA, loop formation is normally unfavorable for those with spacer lengths shorter than the DNA persistence length (approximately 150 bp), because the curvature energy required for forming such small loops becomes too great. For such short loops to occur specific features like intrinsic static bending or binding of an additional protein inducing bending are required. In the case of pLS20, in which the operators O_I_ and O_II_ are separated by only 75 bp, we show that the spacer region contains a static bent.

The first experimental demonstration that a DNA loop can play a crucial role in transcriptional regulation was reported for the *E. coli ara* operon in 1984 [39]. Since then, some other operons have been shown to be also regulated by transcriptional regulator-mediated DNA looping [for reviews see], [ 38], [40]–[43], though the actual number of transcriptional systems for which DNA looping has been conclusively demonstrated is remarkably low. In the case of plasmids, reports demonstrating DNA looping systems are limited to only few cases. One of these includes regulation of initiation of DNA replication at the beta origin of the *E. coli* R6K plasmid [44]; and in the case of *Enterococcus faecalis* plasmid pCF10 it has been proposed that regulation of its conjugation system involves DNA looping mediated by the pheromone-responsive transcriptional regulator PrgX [for review see, 45]. Bio-informatic analyses suggest that DNA looping mediated regulation of transcription is likely to be more common than the few cases for which this has been demonstrated so far. For instance, Cournac and Plumbridge [38] have screened the *E. coli* genome for the presence of putative “simple DNA looping systems” in which looping would involve a single regulator (i.e., this analysis included only transcriptional regulators for which the operator sequence is known, and did not take into account the putative loops that would involve heterologous proteins and/or global transcriptional regulators). Under these restrictive settings, this survey identified 48 genes/operons in which DNA looping mediated regulation is likely to play a role. Interestingly, fourteen of them involve divergently oriented promoters. In the context of our studies, it is worth mentioning the regulation of the conjugation genes located on the integrative and conjugative element ICE*Bs1* that is present in several *B. subtilis* strains. The gene encoding the transcriptional regulator ImmR, and the excision and conjugation genes are expressed from two divergently oriented promoters that are separated by ∼130 bp. At low concentrations, the ImmR protein can bind to six regions, three being proximal to each promoter. It has been suggested that repression of the *imm*R promoter might involve cooperative interactions between ImmR molecules bound to binding sites proximal to both promoters, i.e. DNA looping [46]. Based on the distribution of the operator sites, DNA looping could also be involved in the transcriptional regulation of the Gram-negative plasmids Ti or IncP-plasmids, where divergent promoters have been shown to be involved in controlling both the replication and transfer functions [47], [48].

What are the benefits of DNA looping in general and for the regulation of the conjugation genes of pLS20 in particular? A major consequence of DNA looping is that it results in a high local concentration of the transcriptional regulator at the right place, which would increase its specificity and affinity [for recent review see, 49]. Often, -and Rco_LS20_ is not an exception-, transcriptional regulators are produced in limited amounts per cell. Low numbers of regulators enhance the possibility of transcriptional fluctuations between individual cells within a population. In addition, the intrinsic stochasticity of transcription, -also referred to as noise-, affects the temporal effectiveness of transcriptional regulation; again this is especially prominent when the number of regulatory proteins involved is low. Recent evidences indicate that DNA looping contributes importantly to controlling temporal transcriptional noise, as well as dampening transcriptional fluctuations between cells within a population [50], [51]. Thus, DNA looping contributes to the tight regulation of promoters especially when levels of transcriptional regulators are low by diminishing stochastic fluctuations in transcription.

For some differentiation processes, cell-to-cell or stochastic variability in levels of transcriptional regulators form the basis for activation of these processes, resulting in different behavior of genetically identical cells within a population [52]–[54]. Examples of these processes are the formation of persister cells, development of natural genetic competence, spore formation and swimming/chaining. It is believed that such a bet-hedging strategy is beneficial for the fitness of the species because there will always be some cells that are prepared to cope with a deteriorating environmental condition that may arise in the near future. However, for other processes, there may not be such an advantage and it would then be important to tightly repress the process at times when conditions for that process are not apt. Conjugation probably is such a process because there is no benefit in activating the conjugation genes when there is no recipient present to receive the plasmid. The fact that the efficiency of pLS20 transfer during growth conditions antithetic to conjugation is below the detection limit (at least six orders of magnitude lower than those observed during optimal conjugation conditions) strongly indicates that conjugation genes are tightly repressed under such conditions. However, the tight repression of conjugation should not compromise the ability of rapidly switching to high expression of the conjugation genes when appropriate conditions occur. In pLS20 this is achieved by the constellation of DNA looping combined with autoregulated expression of Rco_LS20_ and overlapping divergent promoters of different strength.

A well-studied genetic switch involving DNA looping is the one that governs the switch from the lysogenic to the lytic state of the *Escherichia coli* phage λ [for review see], [ 55,56]. In the lysogenic or prophage state, phage λ replicates passively with the host while the lytic genes are repressed. This prophage state is extremely stable and can be maintained for many generations. Upon induction of the SOS response, however, a switch is made to the lytic cycle resulting in excision of the phage genome, followed by its amplification and eventually lysis of the cell and release of phage progeny. The early lytic phage λ genes are located in two divergently oriented operons, which are controlled by the lytic promoters *P_R_* and *P_L_*. A third operon, which encodes amongst others the CI transcriptional regulator, is located in between the two early lytic operons such that the promoter of gene *cI*, *P_RM_*, flanks the divergently oriented *P_R_* promoter driving expression of one of the two early operons. In several aspects, functional analogies exist between CI and Rco_LS20_ although they share only 16% of identity at their primary protein sequence level. Both Rco_LS20_ and CI stimulate and repress their own promoter at low and high concentrations, respectively, resulting in a self-sustaining positive feedback loop while keeping the transcriptional regulator in a low concentration range. Above, arguments have been given that for pLS20 this situation, together with the effects of the DNA loop, is important for the tight repression of the *P_c_* promoter during conditions in which conjugation is not favourable, while maintaining the sensitivity to be able to respond rapidly to switch on the conjugation genes when appropriate conditions occur. The transcriptional regulation of λ appears to serve a similar purpose. Thus, on the one hand the lytic genes are tightly repressed since spontaneous switching to the lytic cycle occurs less than once every 10^8^ generations [57]. On the other hand, mutations that specifically eliminate the negative autoregulation of *cI* expression impair prophage induction [58], [59]. Another analogy between the pLS20 and λ systems is that both the regulators Rco_LS20_ and CI, can form higher order oligomers, permitting them to bind cooperatively to multiple sites distributed in two operators, effectively resulting in DNA looping which plays an important role in the genetic regulation of the conjugation and the lytic operon, respectively. Taking the analogy further, it is interesting to note that these regulatory systems both control a process of horizontal gene transfer.

However, there are also several differences between the two systems. For instance, whereas regulation of pLS20 conjugation genes involves a short loop of 75 bp, regulation of the λ lytic genes involves a long loop of 2.3 kb. A second difference is that CI protein forms dimers in solution. A pair of CI dimers tetramerizes when binding to the binding sites in one operator and another dimer pair does the same when binding to the other operator. Upon DNA looping, interaction between the two tetramers constitutes a functional octamer. In addition, when a loop is formed another pair of dimers may bind to additional binding sites present in both operators, and this additional bridge is responsible for repressing *P_RM_* promoter. At present, we do not have such detailed insights in transcriptional regulations at the molecular level for Rco_LS20_. However, instead of dimers, Rco_LS20_ forms tetramers in solution, which probably means that the molecular mechanism by which the pLS20 promoters *P_r_* and *P_c_* are regulated is distinct from the way CI regulates λ promoters *P_R_* and *P_RM_*. Another argument supporting this assumption is the different configuration of the divergent promoters and the binding sites for the regulator protein. In pLS20, the position of promoters *P_c_*/*P_r_* overlaps and the Rco_LS20_ binding sites in O_II_ overlap and flank these core promoters. In λ the binding sites for CI regulator in one operator overlap the *P_R_* promoter and are located upstream of the *P_RM_* core promoter sequences. Finally, a major difference between the DNA looping involved systems of pLS20 and λ is how the switches are induced. In λ, the switch is induced by an SOS response which results in RecA-mediated CI autocleavage. In the case of pLS20, the switch is dictated ultimately by intercellular quorum sensing signaling involving the signaling peptide Phr*_LS20_ that regulates the activity of Rap_LS20_, the anti-repressor of Rco_LS20_ [29]. This quorum sensing system will lead to activation of the conjugation genes when donor cells are surrounded by recipient cells. However, high levels of Phr*_LS20_ will build up when the majority of the cells that surround a donor cell already contain pLS20, and this will inactivate Rap_LS20_ and hence block activation of the conjugation genes.

Besides those described here, it is possible that the pLS20 conjugation genes are regulated by additional mechanism(s). For example, the conspicuously long 5′ untranslated region upstream of gene *28* is predicted to form complex secondary structures, which might modulate expression of the downstream genes in a variety of scenarios. Currently, a study to elucidate a possible role of this long 5′ untranslated region is carried out in our laboratory.

In summary, in this work we have provided evidence that regulation of the conjugation genes present on pLS20 is based on a unique genetic switch that combines at least three levels of control. These include (i) overlapping divergent promoters of different strengths, (ii) auto-stimulation and repression of the weak *P_r_* promoter by the transcriptional regulator at low and elevated concentrations, respectively, combined with simultaneous repression of the divergent strong conjugation promoter, and (iii) DNA looping mediated by binding of Rco_LS20_ regulator to two operators separated by a short loop. Most likely, the combination of these different layers causes tight repression of the main conjugation promoter *P_c_* when conditions for conjugation are not optimal, while allowing the system to switch rapidly to high expression of the conjugation genes when appropriate conditions occur.

## Materials and Methods

### Bacterial strains, plasmids, media and oligonucleotides

Bacterial strains were grown in LB liquid medium or on 1.5% LB agar plates [60]. When appropriate, the following antibiotics were added to media or plates: ampicillin (100 µg/ml), erythromycin (1 and 150 µg/ml in *B. subtilis* and *E. coli*, respectively), chloramphenicol (5 µg/ml), spectinomycin (100 µg/ml), and kanamycin (10 µg/ml). Table S1 lists the *B. subtilis* strains used. All of them are isogenic with *B. subtilis* strain 168. Plasmids and oligonucleotides used are listed in Table S2 and S3, respectively. All oligos were purchased from Isogen Life Science, The Netherlands.

### Transformation

*E. coli* cells were transformed using standardized methods as described in Singh *et al* [61]. For standard *B. subtilis* transformations, competent cells were prepared as described by Bron [62]. Transformants were selected on LB agar plates with appropriate antibiotics.

### Construction of plasmids and strains

Standard molecular methods were used to manipulate DNA [60]. Sequence analysis was used to verify the correctness of all constructs. The same strategy was used to construct *B. subtilis* strains containing a copy of *lacZ* fused to the entire or part of the *rco_LS20_*-gene *28* intergenic DNA region. First, the region of DNA to be cloned was amplified using appropriate primers (see Table S3), purified, and digested with the appropriate restriction enzymes. Next, the fragment was used to prepare a ligation mixture together with the integration vector pDG1663 digested with the same enzymes. The ligation mixture was transformed into *E. coli* XL1-blue cells. The plasmid content of several ampicillin resistant transformants was checked and clones containing the insert with appropriate size and orientation were subjected to DNA sequencing to verify the absence of mutations. The names of the pDG1663 derivatives and their characteristics are listed in Table S2. Plasmid DNA of each pDG1663 derivative was used to transform competent *B. subtilis* 168 cells. Transformants were initially selected for resistance to erythromycin. Next, double cross-over events were distinguished from single cross-over events by selecting transformants sensitive to spectinomycin. The resulting *B. subtilis* strains containing a single copy of *lacZ* preceded by different regions of the *rco_LS20_*-gene *28* region at the *thrC* locus of the *B. subtilis* chromosome are listed in Table S1. Next, plasmid pLS20cat was introduced into the different *lacZ* fusion strains by conjugation. *B. subtilis* strain PKS9 contains a single copy of the *rco_LS20_* gene under the control of the IPTG-inducible *P_spank_* promoter at its *amyE* locus and this cassette is linked to the spectinomycin gene. Chromosomal DNA of strain PKS9 was used to transform competent cells of the various *lacZ* fusion strains in order to construct derivatives of the *lacZ* fusion strains containing the *P_spank_*-*rco_LS20_* cassette.

The following strategy was used to construct a translational fusion of *rco_LS20_* with his_(6)_. The *rco_LS20_* gene was amplified from pLS20cat by PCR using primers oPKS14N and oPKS8. The purified PCR product was digested with *Nco*I and *Sal*I and cloned into the vector pET28b+ digested with the same restriction enzymes to produce plasmid pRco_LS20_-His. *B. subtilis* strain GR90 contains the *rco_LS20_-his*_(6)_ under the control of the *P_spank_* promoter at the *amyE* locus. To construct this strain *rco_LS20_-his*_(6)_ was amplified from pRco_LS20_-His by PCR using primers oGR3 and oGR4. The PCR product was digested with *Nhe*I and *Sph*I and cloned into the vector pDR110 digested with the same enzymes to generate p*P_spank_*rco_LS20_-His. This plasmid was used to transform competent *B. subtilis* cells selecting for spectinomycin resistance. Double cross-over events were selected by loss of amylase gene.

### β-Galactosidase activity assays

β-galactosidase activities were determined as described previously [63]. Overnight grown cultures were diluted 100 times into fresh prewarmed medium and samples were taken every 45 min.

### Conjugation assays

Conjugation was carried out in liquid medium as described previously [29]. The effect of ectopic expression on conjugation of a gene controlled by the IPTG-inducible *P_spank_* promoter was studied as follows. Overnight cultures were diluted in prewarmd LB supplemented with IPTG at the indicated concentrations to an OD_600_ of ∼0.05. Next, samples were taken at regular intervals to determine OD_600_ and were subjected to matings with proper recipient cells.

### RNA isolation and RNA sequencing

Preparation of total RNA samples, RNA sequencing and Bioinformatic analysis of RNAseq data was done as described previously [29].

### Rco_LS20_-His_(6)_ purification

*E. coli* BL21 (DE3) cells carrying plasmid p*rco*_LS20_-His were used to inoculate 1 litre of fresh LB medium supplemented with 30 mg/ml kanamycin and grown at 37°C with shaking. At an OD_600_ of 0.4, expression of *rco_LS20_-his_(6)_* was induced by adding IPTG to a final concentration of 1 mM and growth was continued for 2 h. Cells were further processed as described previously [28]. Purified protein (>95% pure) was dialysed against buffer B (20 mM Tris-HCl pH 8.0, 1 mM EDTA, 250 mM NaCl, 10 mM MgCl_2_, 7 mM β-mercaptoethanol, 50% v/v glycerol) and stored in aliquots at −80°C. Bradford assay was used to determine the protein concentrations.

### Gel retardation

In essence, the gel retardation assays were carried out as described before [28]. Thus, different fragments of intergenic regions between *gene 28* and *rco_LS20_* were amplified by PCR using pLS20cat as template. The resulting PCR fragments were purified and equal concentrations (300 nM) were incubated on ice in binding buffer [20 mM Tris HCl pH 8, 1 mM EDTA, 5 mM MgCl_2_, 0.5 mM DTT, 100 mM KCl, 10% (v/v) glycerol, 0.05 mg ml^−1^ BSA] without and with increasing amounts of purified Rco_LS20_His_(6)_ in a total volume of 16 µl. After careful mixing, samples were incubated for 20 min at 30°C, placed back on ice for 10 min, then loaded onto 2% agarose gel in 0.5XTBE. Electrophoresis was carried out in 0.5X TBE at 50 V at 4°C.Finally, the gel was stained with ethidium bromide, destained in 0.5XTBE and photographed with UV illumination.

### Primer extension experiments

Determination of the transcription start sites by primer extension was performed essentially as described [64]. In brief, total RNA (30 µg) was mixed with 4 pmol of end-labeled oligonucleotide that served as primer; the mixture was heated at 70°C for 5 min and allowed to anneal for 5 min at 23°C. The annealed RNA was ethanol precipitated, resuspended and primer extension was performed with 30 U of AMV reverse transcriptase (Promega) at 42°C, as recommended by the supplier. The extended cDNA products were analysed by electrophoresis on a denaturing 6% urea-polyacrylamide gel, in parallel with a DNA sequence ladder performed by chemical sequencing [65] of a DNA fragment encompassing the mapped promoters (see below). The primer used to map promoter *P_c_* was 5′-ttctagttctttttacac, while that used for promoter *P_r_* was 5′-tctctattgcccacttat. Oligonucleotides were end-labeled with [γ-^32^P]-ATP and T4 polynucleotide kinase as recommended by the supplier (New England Biolabs). The 186 bp DNA fragment that served as sequence ladder was PCR amplified with primers 5′-acggtctagcgcttacaat and 5′-ttctagttctttttacac, the last one labeled at its 5′ end.

### DNase I footprinting

DNaseI footprinting assay was carried out as described [66]. The *P_c_*/*p_r_* promoter encompassing region was amplified by PCR using primers p28_Δ16 and Prom28UpBam, and pLS20cat as template. One of the ends was radio-labeled by digesting the fragment with *Bam*HI and subsequently filling in the end with exo^−^ Klenow fragment in the presence of [α-^32^P]-ATP.

### Computer-assisted analysis

Presence of conserved motifs was searched by using motif-identification programs MEME [30] and BIOPROSPECTOR [31]. Prediction of the static bending properties of DNA sequences was carried out by calculating the global 3D structure according to the dinucleotide wedge model [67]. All graphics work was done by using Adobe Photoshop CS2 and adobe illustrator. Graphs were plotted using Excel program.

### Ultracentrifugation

Sedimentation velocity assay. Samples in 20 mM Tris-HCl, 250 mM NaCl, 10 mM MgCl_2_, 1 mM EDTA and 100 mM glycerol, pH 7.4, were loaded (320 µL) into analytical ultracentrifugation cells. The experiments were carried out at 43–48 krpm in an XL-I analytical ultracentrifuge (Beckman-Coulter Inc.) equipped with UV-VIS absorbance and Raleigh interference detection systems. Sedimentation profiles were recorded at 280 nm. Sedimentation coefficient distributions were calculated by least-squares boundary modelling of sedimentation velocity data using the continuous distribution c(s) Lamm equation model as implemented by SEDFIT 14.1 [68]. Experimental *s* values were corrected to standard conditions (water, 20°C, and infinite dilution) using the program SEDNTERP [69] to get the corresponding standard *s* values (*s*_20,*w*_).

Sedimentation equilibrium assay. Using the same experimental conditions as in the SV experiments, short columns (90 µL) SE experiments were carried out at speeds ranging from 7,000 to 10,000 rpm and at 280 nm. After the last equilibrium scan, a high-speed centrifugation run (48,000 rpm) was done to estimate the corresponding baseline offsets. Weight-average buoyant molecular weights of protein were determined by fitting a single species model to the experimental data using the HeteroAnalysis program [70], and corrected for solvent composition and temperature with the program SEDNTERP [69].

## Supporting Information

Figure S1 The *rco_LS20_* – gene *28* intergenic region contains a strong promoter that is inhibited by the pLS20cat encoded protein Rco_LS20_. Strains were streaked on Xgal-containing LB plates and incubated for 16 hours at 37°C. When indicated, plates were also supplemented with 10 µM IPTG in the case of PKS5. Strain PKS3 contains a cassette at the *thrC* locus in which the *lacZ* gene is preceded by the 570 bp rco_LS20_-gene *28* intergenic region (sequences in between the ribosomal binding sites of the divergently oriented genes *28* and *rco_LS20_*). PKS8 is a derivative of PKS3 harboring pLS20cat. PKS5 is a derivative of PKS3 containing the *P_spank_*-*rco_LS20_* cassette at *amy*E. The negative control strain PKS7 contains a promoterless version of *lac*Z at the *thr*C locus.
(TIF)

Figure S2 The 75 bp region separating operators O_I_ and O_II_ is predicted to contain a static bent. The global 3D structure of a 256 bp DNA region encompassing operators O_I_ and O_II_ was predicted according the dinucleotide wedge mode using the online webpage http://www.lfd.uci.edu/~gohlke/dnacurve/. For clarity, sequences corresponding to promoters *P_c_*/*P_r_* and motifs in operators O_I_ and O_II_ are presented as space filling. Positions of the promoters and Rco_LS20_ binding motifs are given in blue and purple respectively.
(TIF)

Figure S3 Enlarging the distance between operators O_I_ and O_II_ with half a helical turn affects Rco_LS20_-mediated inhibition of promoter *P_c_*. Strains containing F_I_c_ and F_I_c_+5 fused to *lacZ* (PKS3 and GR189, respectively) and their derivatives harboring pLS20cat (PKS8 and GR191, respectively) were spread on Xgal-containing LB agar plates and photographed after 24 hours incubation at 37°C.
(TIF)

Table S1 Strains used.
(DOCX)

Table S2 Plasmids used.
(DOCX)

Table S3 Oligonucleotides used.
(DOCX)

## References

[1] Ochman H, Lawrence JG, Groisman EA (2000) Lateral gene transfer and the nature of bacterial innovation. Nature 405: 299–304. 10830951 10.1038/35012500

[2] Frost LS, Leplae R, Summers AO, Toussaint A (2005) Mobile genetic elements: the agents of open source evolution. Nat Rev Micobiol 3: 722–732. 10.1038/nrmicro1235 16138100

[3] Thomas CM, Nielsen KM (2005) Mechanisms of, and barriers to, horizontal gene transfer between bacteria. Nat Rev Microbiol 3: 711–721. 16138099 10.1038/nrmicro1234

[4] Novick RP, Christie GE, Penades JR (2010) The phage-related chromosomal islands of Gram-positive bacteria. Nat Rev Microbiol 8: 541–551. 20634809 10.1038/nrmicro2393 PMC3522866

[5] Wozniak RA, Waldor MK (2010) Integrative and conjugative elements: mosaic mobile genetic elements enabling dynamic lateral gene flow. Nat Rev Microbiol 8: 552–563. 20601965 10.1038/nrmicro2382

[6] Auchtung JM, Lee CA, Monson RE, Lehman AP, Grossman AD (2005) Regulation of a Bacillus subtilis mobile genetic element by intercellular signaling and the global DNA damage response. Proc Natl Acad Sci U S A 102: 12554–12559. 16105942 10.1073/pnas.0505835102 PMC1194945

[7] Frost LS, Koraimann G (2010) Regulation of bacterial conjugation: balancing opportunity with adversity. Future Microbiol 5: 1057–1071. 20632805 10.2217/fmb.10.70

[8] Smillie C, Garcillán-Barcia MP, Francia MV, Rocha EPC, De la Cruz F (2010) Mobility of plasmids. Microbiol Mol Biol Rev 74: 434–452. 20805406 10.1128/MMBR.00020-10 PMC2937521

[9] Alvarez-Martinez CE, Christie PJ (2009) Biological diversity of prokaryotic type IV secretion systems. Microbiol Mol Biol Rev 73: 775–808. 19946141 10.1128/MMBR.00023-09 PMC2786583

[10] Fronzes R, Christie PJ, Waksman G (2009) The structural biology of type IV secretion systems. Nat Rev Microbiol 7: 703–714. 19756009 10.1038/nrmicro2218 PMC3869563

[11] Goessweiner-Mohr N, Grumet L, Arends K, Pavkov-Keller T, Gruber CC, Gruber K, Birner-Gruenberger R, Kropec-Huebner A, Huebner J, Grohmann E, Keller W (2013) The 2.5 A structure of the enterococcus conjugation protein TraM resembles VirB8 type IV secretion proteins. J Biol Chem 288: 2018–2028. 23188825 10.1074/jbc.M112.428847 PMC3548508

[12] Li J, Adams V, Bannam TL, Miyamoto K, Garcia JP, Uzal FA, Rood JI, McClane BA (2013) Toxin plasmids of Clostridium perfringens. Microbiol Mol Biol Rev 77: 208–233. 23699255 10.1128/MMBR.00062-12 PMC3668675

[13] Liu MA, Kwong SM, Jensen SO, Brzoska AJ, Firth N (2013) Biology of the staphylococcal conjugative multiresistance plasmid pSK41. Plasmid 70: 42–51. 23415796 10.1016/j.plasmid.2013.02.001

[14] Caryl JA, O'Neill AJ (2009) Complete nucleotide sequence of pGO1, the prototype conjugative plasmid from the Staphylococci. Plasmid 62: 35–38 S. 19298835 10.1016/j.plasmid.2009.03.001

[15] Clewell DB (2011) Tales of conjugation and sex pheromones: A plasmid and enterococcal odyssey. Mob Genet Elements 1: 38–54. 22016844 10.4161/mge.1.1.15409 PMC3190283

[16] Dunny GM, Johnson CM (2011) Regulatory circuits controlling enterococcal conjugation: lessons for functional genomics. Curr Opin Microbiol 14: 174–180. 21353627 10.1016/j.mib.2011.01.008 PMC3144862

[17] Chatterjee A, Cook LC, Shu CC, Chen Y, Manias DA, Ramkrishna D, Dunny GM, Hu WS (2013) Antagonistic self-sensing and mate-sensing signaling controls antibiotic-resistance transfer. Proc Natl Acad Sci U S A 110: 7086–7090. 23569272 10.1073/pnas.1212256110 PMC3637703

[18] Sonenshein, A. L., Hoch, J. A., and Losick, R. (1993) *Bacillus subtilis* and other Gram-positive bacteria; Biochemistry, physiology, and molecular genetics. Washington, D.C.: American Society for Microbiology. 987 p.

[19] Sonenshein, A. L., Hoch, J. A., and Losick, R. (2001) *Bacillus subtilis* and its closest relatives: from genes to cells. ASM Press.

[20] Cutting SM (2011) Bacillus probiotics. Food Microbiol 28: 214–220. 21315976 10.1016/j.fm.2010.03.007

[21] Tanaka T, Koshikawa T (1977) Isolation and characterization of four types of plasmids from *Bacillus subtilis (natto)* . J Bacteriol 131: 699–701. 407218 10.1128/jb.131.2.699-701.1977 PMC235484

[22] Koehler TM, Thorne CB (1987) *Bacillus subtilis (natto)* plasmid pLS20 mediates interspecies plasmid transfer. J Bacteriol 169: 5271–5278. 3117774 10.1128/jb.169.11.5271-5278.1987 PMC213936

[23] Itaya M, Sakaya N, Matsunaga S, Fujita K, Kaneko S (2006) Conjugational transfer kinetics of pLS20 between Bacillus subtilis in liquid medium. Biosci Biotechnol Biochem 70: 740–742. 16556997 10.1271/bbb.70.740

[24] Bauer T, Rosch T, Itaya M, Graumann PL (2011) Localization pattern of conjugation machinery in a Gram-positive bacterium. J Bacteriol 193: 6244–6256. 21949064 10.1128/JB.00175-11 PMC3209232

[25] Rösch TC, Golman W, Hucklesby L, Gonzalez-Pastor JE, Graumann PL (2014) The presence of conjugative plasmid pLS20 affects global transcription of Its Bacillus subtilis host and confers beneficial stress resistance to cells. Appl Environ Microbiol 80: 1349–1358. 24334659 10.1128/AEM.03154-13 PMC3911045

[26] Meijer WJJ, de Boer A, van Tongeren S, Venema G, Bron S (1995) Characterization of the replication region of the *Bacillus subtilis* plasmid pLS20: a novel type of replicon. Nucleic Acids Res 23: 3214–3223. 7667098 10.1093/nar/23.16.3214 PMC307180

[27] Derman AI, Becker EC, Truong BD, Fujioka A, Tucey TM, Erb ML, Patterson PC, Pogliano J (2009) Phylogenetic analysis identifies many uncharacterized actin-like proteins (Alps) in bacteria: regulated polymerization, dynamic instability and treadmilling in Alp7A. Mol Microbiol 73: 534–552. 19602153 10.1111/j.1365-2958.2009.06771.x PMC2814180

[28] Singh PK, Ramachandran G, Duran-Alcalde L, Alonso C, Wu LJ, Meijer WJ (2012) Inhibition of Bacillus subtilis natural competence by a native, conjugative plasmid-encoded comK repressor protein. Environ Microbiol 14: 2812–2825. 22779408 10.1111/j.1462-2920.2012.02819.x

[29] Singh PK, Ramachandran G, Ramos-Ruiz R, Peiro-Pastor R, Abia D, Wu LJ, Meijer WJ (2013) Mobility of the Native Bacillus subtilis Conjugative Plasmid pLS20 Is Regulated by Intercellular Signaling. PLoS Genet 9: e1003892. 24204305 10.1371/journal.pgen.1003892 PMC3814332

[30] Bailey TL, Elkan C (1994) Fitting a mixture model by expectation maximization to discover motifs in biopolymers. Proc Int Conf Intell Syst Mol Biol 2: 28–36. 7584402

[31] Liu X, Brutlag DL, Liu JS (2001) BioProspector: discovering conserved DNA motifs in upstream regulatory regions of co-expressed genes. Pac Symp Biocomput 127–138. 11262934

[32] Hagerman PJ (1988) Flexibility of DNA. Annu Rev Biophys Biophys Chem 17: 265–286. 3293588 10.1146/annurev.bb.17.060188.001405

[33] Haran TE, Mohanty U (2009) The unique structure of A-tracts and intrinsic DNA bending. Q Rev Biophys 42: 41–81. 19508739 10.1017/S0033583509004752

[34] Beck CF, Warren RA (1988) Divergent promoters, a common form of gene organization. Microbiol Rev 52: 318–326. 3054465 10.1128/mr.52.3.318-326.1988 PMC373147

[35] Wang P, Yang J, Lawley B, Pittard AJ (1997) Repression of the aroP gene of Escherichia coli involves activation of a divergent promoter. J Bacteriol 179: 4213–4218. 9209035 10.1128/jb.179.13.4213-4218.1997 PMC179241

[36] Wang P, Yang J, Ishihama A, Pittard AJ (1998) Demonstration that the TyrR protein and RNA polymerase complex formed at the divergent P3 promoter inhibits binding of RNA polymerase to the major promoter, P1, of the aroP gene of Escherichia coli. J Bacteriol 180: 5466–5472. 9765583 10.1128/jb.180.20.5466-5472.1998 PMC107600

[37] Bendtsen KM, Erdossy J, Csiszovszki Z, Svenningsen SL, Sneppen K, Krishna S, Semsey S (2011) Direct and indirect effects in the regulation of overlapping promoters. Nucleic Acids Res 39: 6879–6885. 21609952 10.1093/nar/gkr390 PMC3167631

[38] Cournac A, Plumbridge J (2013) DNA looping in prokaryotes: experimental and theoretical approaches. J Bacteriol 195: 1109–1119. 23292776 10.1128/JB.02038-12 PMC3591992

[39] Dunn TM, Hahn S, Ogden S, Schleif RF (1984) An operator at −280 base pairs that is required for repression of araBAD operon promoter: addition of DNA helical turns between the operator and promoter cyclically hinders repression. Proc Natl Acad Sci U S A 81: 5017–5020. 6089170 10.1073/pnas.81.16.5017 PMC391628

[40] Adhya S (1989) Multipartite genetic control elements: communication by DNA loop. Annu Rev Genet 23: 227–250. 2694932 10.1146/annurev.ge.23.120189.001303

[41] Matthews KS (1992) DNA looping. Microbiol Rev 56: 123–136. 1579106 10.1128/mr.56.1.123-136.1992 PMC372857

[42] Schleif R (1992) DNA looping. Annu Rev Biochem 61: 199–223. 1497310 10.1146/annurev.bi.61.070192.001215

[43] Hochschild A, Lewis M (2009) The bacteriophage lambda CI protein finds an asymmetric solution. Curr Opin Struct Biol 19: 79–86. 19181516 10.1016/j.sbi.2008.12.008 PMC2684985

[44] Mukherjee S, Erickson H, Bastia D (1988) Enhancer-origin interaction in plasmid R6K involves a DNA loop mediated by initiator protein. Cell 52: 375–383. 3345564 10.1016/s0092-8674(88)80030-8

[45] Dunny GM (2013) Enterococcal sex pheromones: signaling, social behavior, and evolution. Annu Rev Genet 47: 457–482. 24050179 10.1146/annurev-genet-111212-133449

[46] Auchtung JM, Lee CA, Garrison KL, Grossman AD (2007) Identification and characterization of the immunity repressor (ImmR) that controls the mobile genetic element ICEBs1 of Bacillus subtilis. Mol Microbiol 64: 1515–1528. 17511812 10.1111/j.1365-2958.2007.05748.x PMC3320793

[47] Zatyka M, Jagura-Burdzy G, Thomas CM (1997) Transcriptional and translational control of the genes for the mating pair formation apparatus of promiscuous IncP plasmids. J Bacteriol 179: 7201–7209. 9393681 10.1128/jb.179.23.7201-7209.1997 PMC179667

[48] Lang J, Faure D (2014) Functions and regulation of quorum-sensing in Agrobacterium tumefaciens. Front Plant Sci 5: 14. 24550924 10.3389/fpls.2014.00014 PMC3907764

[49] Oehler S, Muller-Hill B (2010) High local concentration: a fundamental strategy of life. J Mol Biol 395: 242–253. 19883663 10.1016/j.jmb.2009.10.056

[50] Vilar JM, Saiz L (2005) DNA looping in gene regulation: from the assembly of macromolecular complexes to the control of transcriptional noise. Curr Opin Genet Dev 15: 136–144. 15797196 10.1016/j.gde.2005.02.005

[51] Saiz L, Vilar JM (2006) DNA looping: the consequences and its control. Curr Opin Struct Biol 16: 344–350. 16714105 10.1016/j.sbi.2006.05.008

[52] Korobkova E, Emonet T, Vilar JM, Shimizu TS, Cluzel P (2004) From molecular noise to behavioural variability in a single bacterium. Nature 428: 574–578. 15058306 10.1038/nature02404

[53] Dubnau D, Losick R (2006) Bistability in bacteria. Mol Microbiol 61: 564–572. 16879639 10.1111/j.1365-2958.2006.05249.x

[54] Veening JW, Smits WK, Kuipers OP (2008) Bistability, epigenetics, and bet-hedging in bacteria. Annu Rev Microbiol 62: 193–210. 18537474 10.1146/annurev.micro.62.081307.163002

[55] Dodd IB, Shearwin KE, Egan JB (2005) Revisited gene regulation in bacteriophage lambda. Curr Opin Genet Dev 15: 145–152. 15797197 10.1016/j.gde.2005.02.001

[56] Oppenheim AB, Kobiler O, Stavans J, Court DL, Adhya S (2005) Switches in bacteriophage lambda development. Annu Rev Genet 39: 409–429. 16285866 10.1146/annurev.genet.39.073003.113656

[57] Little JW, Shepley DP, Wert DW (1999) Robustness of a gene regulatory circuit. EMBO J 18: 4299–4307. 10428968 10.1093/emboj/18.15.4299 PMC1171506

[58] Dodd IB, Perkins AJ, Tsemitsidis D, Egan JB (2001) Octamerization of lambda CI repressor is needed for effective repression of P(RM) and efficient switching from lysogeny. Genes Dev 15: 3013–3022. 11711436 10.1101/gad.937301 PMC312832

[59] Dodd IB, Shearwin KE, Perkins AJ, Burr T, Hochschild A, Egan JB (2004) Cooperativity in long-range gene regulation by the lambda CI repressor. Genes Dev 18: 344–354. 14871931 10.1101/gad.1167904 PMC338286

[60] Sambrook, J., Fritsch, E. F., and Maniatis, T. (1989) Molecular cloning: a laboratory manual. Cold Spring Harbor, New York: Cold Spring Harbor Laboratory Press.

[61] Singh PK, Ballestero-Beltran S, Ramachandran G, Meijer WJ (2010) Complete nucleotide sequence and determination of the replication region of the sporulation inhibiting plasmid p576 from Bacillus pumilus NRS576. Res Microbiol 161: 772–782. 20863889 10.1016/j.resmic.2010.07.007

[62] Bron S (1990) Plasmids. In: Harwood CR, Cutting SM, editors. Molecular Biological Methods for *Bacillus*. Chichester, UK: John Wiley & Sons Ltd. pp. 75–174.

[63] Miller, J. H. (1982) Experiments in molecular genetics. Cold Spring Harbor, New York: Cold Spring Harbor Laboratory Press.

[64] Moreno R, Fonseca P, Rojo F (2012) Two small RNAs, CrcY and CrcZ, act in concert to sequester the Crc global regulator in Pseudomonas putida, modulating catabolite repression. Mol Microbiol 83: 24–40. 22053874 10.1111/j.1365-2958.2011.07912.x

[65] Maxam AM, Gilbert W (1980) Sequencing end-labeled DNA with base-specific chemical cleavages. Methods Enzymol 65: 499–560. 6246368 10.1016/s0076-6879(80)65059-9

[66] Meijer WJJ, Castilla-Llorente V, Villar L, Murray H, Errington J, Salas M (2005) Molecular basis for the exploitation of spore formation as survival mechanism by virulent phage φ29. EMBO J 24: 3647–3657. 16193065 10.1038/sj.emboj.7600826 PMC1276709

[67] Bolshoy A, McNamara P, Harrington RE, Trifonov EN (1991) Curved DNA without A-A: experimental estimation of all 16 DNA wedge angles. Proc Natl Acad Sci U S A 88: 2312–2316. 2006170 10.1073/pnas.88.6.2312 PMC51221

[68] Schuck P (2000) Size-distribution analysis of macromolecules by sedimentation velocity ultracentrifugation and lamm equation modeling. Biophys J 78: 1606–1609. 10692345 10.1016/S0006-3495(00)76713-0 PMC1300758

[69] Laue TM, Shah BD, Ridgeway TM, Pelletier SL (1992) Interpretation of analytical sedimentation data for proteins. In: Harding SE, Rowe AJ, Horton JC, editors. Analytical ultracentrifugation in biochemistry and polymer science. Cambridge, UK: Royal Society of Chemistry. pp. 90–125.

[70] Cole JL (2004) Analysis of heterogeneous interactions. Methods Enzymol 384: 212–232. 15081689 10.1016/S0076-6879(04)84013-8 PMC2924680

[71] Shimomaye E, Salvato M (1989) Use of avian myeloblastosis virus reverse transcriptase at high temperature for sequence analysis of highly structured RNA. Gene Anal Tech 6: 25–28. 2473018 10.1016/0735-0651(89)90022-8

[72] Loreau N, Boiziau C, Verspieren P, Shire D, Toulme JJ (1990) Blockage of AMV reverse transcriptase by antisense oligodeoxynucleotides. FEBS Lett 274: 53–56. 1701402 10.1016/0014-5793(90)81327-k

[73] Crooks GE, Hon G, Chandonia JM, Brenner SE (2004) WebLogo: a sequence logo generator. Genome Res 14: 1188–1190. 15173120 10.1101/gr.849004 PMC419797

