# Supplementary figures and images for: A Complex Genetic Switch Involving Overlapping Divergent Promoters and DNA Looping Regulates Expression of Conjugation Genes of a Gram-positive Plasmid
Source: PLoS Genet. 2014 Oct 23;10(10):e1004733. doi: 10.1371/journal.pgen.1004733 (PMC4207663; doi:10.1371/journal.pgen.1004733)

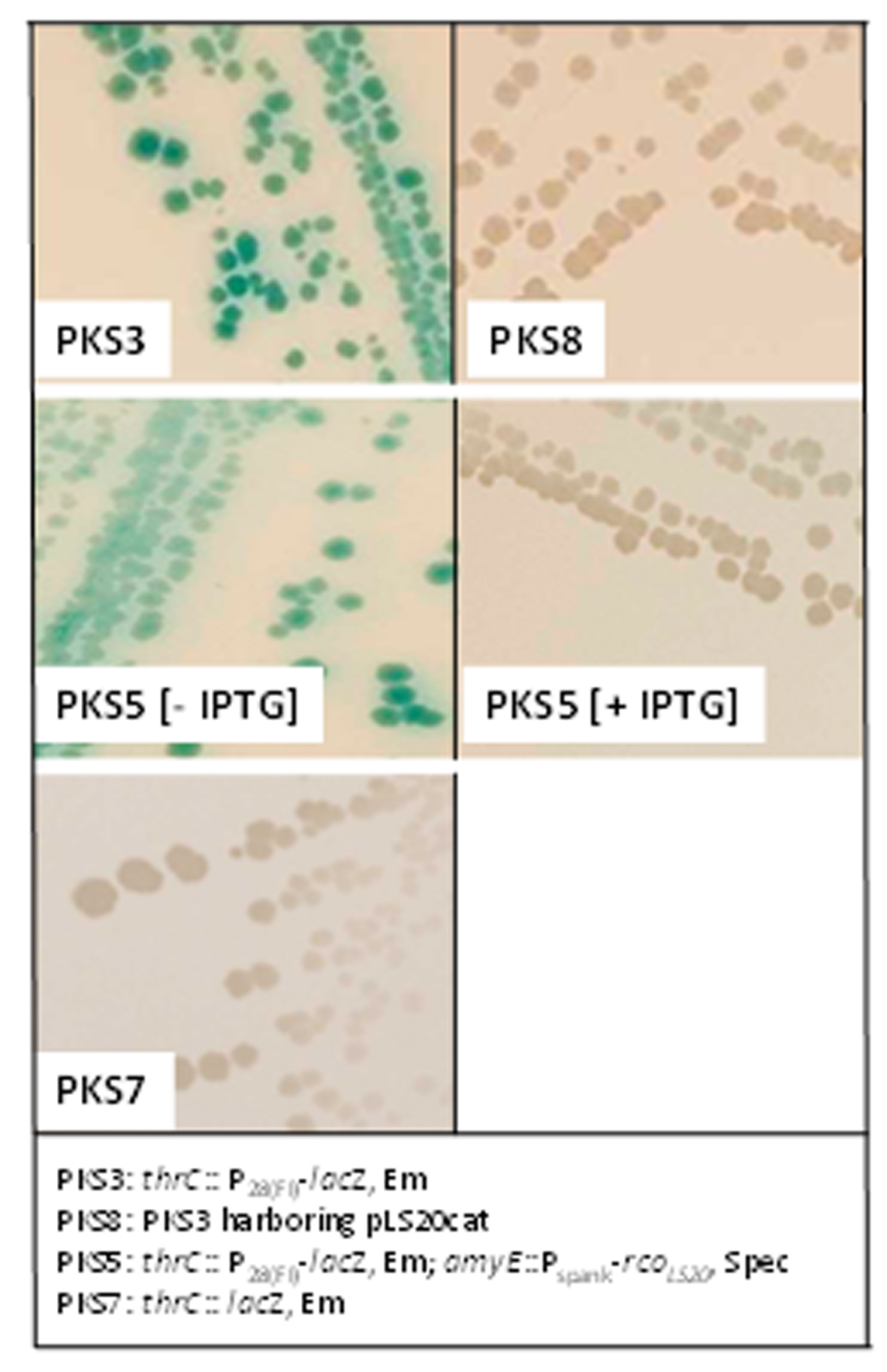

Supplement: Figure S1 — The rcoLS20 – gene 28 intergenic region contains a strong promoter that is inhibited by the pLS20cat encoded protein RcoLS20. Strains were streaked on Xgal-containing LB plates and incubated for 16 hours at 37°C. When indicated, plates were also supplemented with 10 µM IPTG in the case of PKS5. Strain PKS3 contains a cassette at the thrC locus in which the lacZ gene is preceded by the 570 bp rcoLS20-gene 28 intergenic region (sequences in between the ribosomal binding sites of the divergently oriented genes 28 and rcoLS20). PKS8 is a derivative of PKS3 harboring pLS20cat. PKS5 is a derivative of PKS3 containing the Pspank-rcoLS20 cassette at amyE. The negative control strain PKS7 contains a promoterless version of lacZ at the thrC locus. (TIF) [file pgen.1004733.s001.tif]

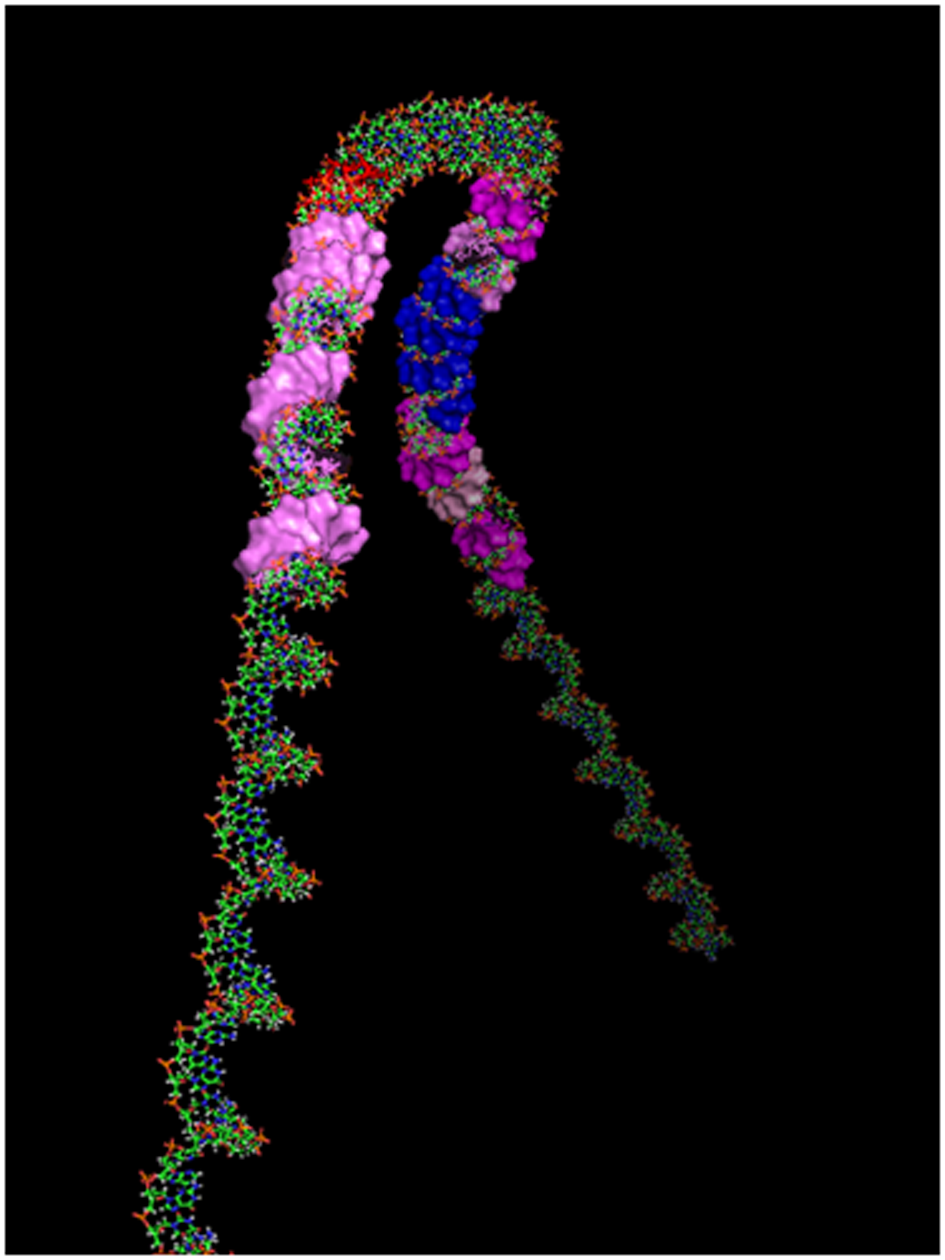

Supplement: Figure S2 — The 75 bp region separating operators OI and OII is predicted to contain a static bent. The global 3D structure of a 256 bp DNA region encompassing operators OI and OII was predicted according the dinucleotide wedge mode using the online webpage http://www.lfd.uci.edu/~gohlke/dnacurve/. For clarity, sequences corresponding to promoters Pc/Pr and motifs in operators OI and OII are presented as space filling. Positions of the promoters and RcoLS20 binding motifs are given in blue and purple respectively. (TIF) [file pgen.1004733.s002.tif]

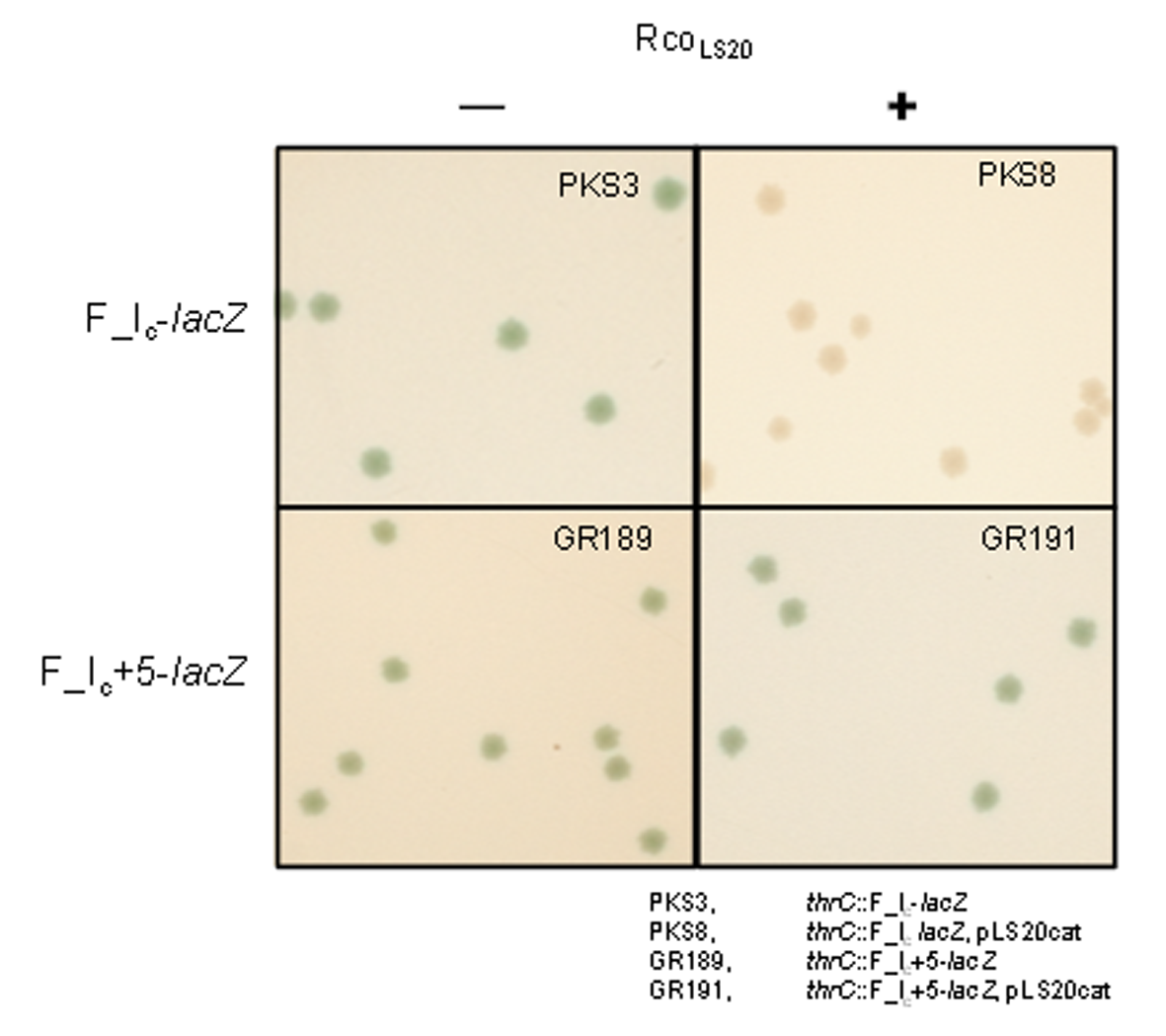

Supplement: Figure S3 — Enlarging the distance between operators OI and OII with half a helical turn affects RcoLS20-mediated inhibition of promoter Pc. Strains containing F_Ic and F_Ic+5 fused to lacZ (PKS3 and GR189, respectively) and their derivatives harboring pLS20cat (PKS8 and GR191, respectively) were spread on Xgal-containing LB agar plates and photographed after 24 hours incubation at 37°C. (TIF) [file pgen.1004733.s003.tif]
