# Supplementary material for: A Complex Genetic Switch Involving Overlapping Divergent Promoters and DNA Looping Regulates Expression of Conjugation Genes of a Gram-positive Plasmid
Source: PLoS Genet. 2014 Oct 23;10(10):e1004733. doi: 10.1371/journal.pgen.1004733 (PMC4207663; doi:10.1371/journal.pgen.1004733)
Supplement: Table S1 — Strains used. (DOCX) [file pgen.1004733.s004.docx]

| **Table S1. Strains used** | | |
| --- | --- | --- |
| **Strains** | **Genotype or description** | **References or source** |
| *E. coli* |  |  |
| XL1-Blue | endA1 gyrA96(nal^R^) thi-1 recA1 relA1 lac glnV44 F'[ ::Tn10 proAB^+^ lacI^q^ Δ(*lacZ*)M15] hsdR17(r_K_^-^ m_K_^+^) | [1] |
| *B. subtilis* |  |  |
| 168 (1A700) | *trpC2* | BGSC* |
| PKS3 | *trpC2*  *thrC*::P_c(F_Ic)_ -*lacZ* (*em*) | This work |
| PKS5 | *trpC2*  *thrC*::P_c(F_Ic)_ -*lacZ*(*em*) *amyE*::P_spank_*rco_LS20_* (*spc*) | This work |
| PKS7 | *trpC2* *thrC*::Δ -*lacZ*(*em*) | This work |
| PKS8 | *trpC2 thrC*::P_c(F_Ic)_-*lacZ*(*em*) containing plasmid pLS20*cat* | This work |
| PKS11 | *trpC2* containing plasmid pLS20*cat* | [2] |
| PKS30 | *trpC2 thrC::*P_c(F_IIIc)_-*lacZ*(*em*) | This work |
| PKS31 | *trpC2 thrC::*P_r(F_IIIr)_-*lacZ*(*em*) | This work |
| PKS32 | *trpC2 thrC*::P_c(F_IIIc)_-*lacZ*(*em*), containing plasmid pLS20*cat* | This work |
| GR9 | *trpC2 thrC::*P_r(F_IIIr)_-*lacZ*(*em*), containing plasmid pLS20*cat* | This work |
| GR10 | *trpC2 thrC::*P_c(F_IIc)_-*lacZ*(*em*) | This work |
| GR11 | *trpC2 thrC::*P_c(F_IIc)_-*lacZ*(*em*), containing plasmid pLS20*cat* | This work |
| GR12 | *trpC2 thrC::*P_c(F_IIc)_-*lacZ*(*em*)  *amyE*::P_spank_*rco_LS20_* (*spc*) | This work |
| GR14 | *trpC2 thrC::*P_r(F_IIIr)_-*lacZ*(*em*)  *amyE*::P_spank_*rco_LS20_* (*spc*) | This work |
| GR16 | *trpC2 thrC::*P_c(F_IIIc)_-*lacZ*(*em*)  amy*E*::P_spank_*rco_LS20_* (*spc*) | This work |
| GR25 | *trpC2 thrC*::P_r(F_Ir)-_*lacZ* (*em*) | This work |
| GR27 | *trpC2 thrC*::P_r(F_IVr)-_*lacZ* (*em*) | This work |
| GR28 | *trpC2 thrC*::P_c(F_IVc)_-*lacZ* (*em*) | This work |
| GR29 | *trpC2 thrC*::P_r(F_Vr)_-*lacZ (em*) | This work |
| GR30 | *trpC2 thrC*::P_c(F_Vc)_-*lacZ* (*em*) | This work |
| GR33 | *trpC2 thrC*::P_r(F_IVr)_-*lacZ* (*em*), containing plasmid pLS20*cat* | This work |
| GR34 | *trpC2 thrC*::P_c(F_IVc)_-*lacZ* (*em*), containing plasmid pLS20*cat* | This work |
| GR35 | *trpC2 thrC*::P_r(F_Vr)_-*lacZ (em*), containing plasmid pLS20*cat* | This work |
| GR36 | *trpC2 thrC*::P_c(F_Vc)_*lacZ* (*em*), containing plasmid pLS20*cat* | This work |
| GR39 | *trpC2 thrC*::P_r(F_Ir)_*lacZ* (*em*), containing plasmid pLS20*cat* | This work |
| GR42 | *trpC2 thrC*::P_c(F_IVc)_*lacZ* (*em*)  *amyE*::P_spank_*rco_LS20_* (*spc*) | This work |
| GR43 | *trpC2 thrC*::P_c(F_Vc)_*lacZ* (*em*)  *amyE*::P_spank_*rco_LS20_* (*spc*) | This work |
| GR62 | *trpC2 thrC*::P_r(F_IAr)_*lacZ* (*em*) | This work |
| GR66 | *trpC2 thrC*::P_r(F_IAr)_*lacZ* (*em*), containing plasmid pLS20*cat* | This work |
| GR68 | *trpC2 thrC*::P_c(F_VIIc)_*lacZ* (*em*) | This work |
| GR69 | *trpC2 thrC*::P_r(F_VIIr)_*lacZ* (*em*) | This work |
| GR70 | *trpC2 thrC*::P_c(F_VIIIc)_*lacZ* (*em*) | This work |
| GR71 | *trpC2 thrC*::P_r(F_VIIIr)_*lacZ* (*em*) | This work |
| GR76 | *trpC2 thrC*::P_r(F_IAr)_*lacZ* (*em*)  amy*E*::P_spank_*rco_LS20_* (*spc*) | This work |
| GR82 | *trpC2 thrC*::P_r(F_VIIr)_*lacZ* (*em*), containing plasmid pLS20*cat* | This work |
| GR83 | *trpC2* *thrC*::Δ *lacZ*(*em*), containing plasmid pLS20*cat* | This work |
| GR90 | trpC2 *amyE*::P*_spank_*rco_LS20_-his | This work |
| GR92 | *trpC2 thrC*::P_r(F_Ir)_-*lacZ* (*em*)  *amyE*::P_spank_*rco_LS20_* (*spc*) | This work |
| GR97 | *trpC2 thrC*::P_r(F_IVr)_-*lacZ* (*em*)  *amyE*::P_spank_*rco_LS20_* (*spc*) | This work |
| GR102 | *trpC2 thrC*::P_r(F_Vr)_-*lacZ* (*em*)  *amyE*::P_spank_*rco_LS20_* (*spc*) | This work |
| GR116 | *trpC2 thrC*::P_r(F_VIIIr)_-*lacZ* (*em*), containing plasmid pLS20*cat* | This work |
| GR164 | *trpC2 thrC*::P_c(F_VIIc)_-*lacZ* (*em*)  amy*E*::P_spank_*rco_LS20_* (*spc*) | This work |
| GR165 | *trpC2 thrC*::P_c(F_VIIc)_-*lacZ* (*em*), containing plasmid pLS20*cat* | This work |
| GR189 | *trpC2 thrC*::P_c(F_Ic+5)_-*lacZ* (*em*) | This work |
| GR191 | *trpC2 thrC*::P_c(F_Ic+5)_-*lacZ* (*em*), containing plasmid pLS20*cat* | This work |
| GR195 | *trpC2 thrC*::P_c(F_Ic+5)_-*lacZ* (*em*)  *amyE*::P_spank_*rco_LS20_* (*spc*) | This work |
| *, BGSC: *Bacillus* Genetic Stock Center, Department of Bioch*em*istry, The Ohio State University, Columbus, OH, USA. (<http://www.bgsc.org/>) | | |

**References**

1. Bullock WO, Fernandez JM, Short JM (1987) XL1-blue: a high efficiency plasmid transforming *recA* *Escherichia coli* strain with Beta-galactosidase selection. Biotechniques 5: 376-379.

2. Singh PK, Ramachandran G, Duran-Alcalde L, Alonso C, Wu LJ, Meijer WJ (2012) Inhibition of Bacillus subtilis natural competence by a native, conjugative plasmid-encoded comK repressor protein. Environ Microbiol 14: 2812-2825. 10.1111/j.1462-2920.2012.02819.x [doi].
