# Supplementary material for: A Complex Genetic Switch Involving Overlapping Divergent Promoters and DNA Looping Regulates Expression of Conjugation Genes of a Gram-positive Plasmid
Source: PLoS Genet. 2014 Oct 23;10(10):e1004733. doi: 10.1371/journal.pgen.1004733 (PMC4207663; doi:10.1371/journal.pgen.1004733)
Supplement: Table S2 — Plasmids used. (DOCX) [file pgen.1004733.s005.docx]

| **Table S2. Plasmids used** | | |
| --- | --- | --- |
| **Plasmids** | **Description** | **Reference or source** |
| pDR110 | *B. subtilis amyE* integration vector containing IPTG-inducible Pspank promoter | D. Rudner * |
| pDG1663 | *B. subtilis* *thrC* integration vector containing promoter-less *lacZ* gene which is used for promoter screening | [3], BGSC |
| pLS20*cat* | Native plasmid pLS20 labelled with Cm resistance cassette in unique SalI site. | [4] |
| pTZ57R/T | T/A cloning vector | Fermentas |
| pDP*_spank_*rco_LS20_ | *rco_LS20_* gene is cloned in integration vector pDR110 | [5] |
| pET28b(+) | Vector for expressing His tagged heterologous proteins in *E. coli* | Novagene, Madison, WI, USA |
| pDGP_c(F_Ic)_ | Fragment I_c_ cloned in front of *lacZ* gene of pDG1663 | This work |
| pDGP_c(F _Ic+5bp)_ | Fragment I_c+5bps_ cloned in front of *l*a*cZ* gene of pDG1663 | This work |
| pDGP_c(F_IIc)_ | Fragment II_c_ cloned in front of *lacZ* gene of pDG1663 | This work |
| pDGP_c(F_IIIc)_ | Fragment III_c_ cloned in front of *lacZ* gene of pDG1663 | This work |
| pDGP_c(F_IVc)_ | Fragment IV_c_ cloned in front of *lacZ* gene of pDG1663 | This work |
| pDGP_c(F_Vc)_ | Fragment V_c_ cloned in front of *lacZ* gene of pDG1663 | This work |
| pDGP_c(F_VIIc)_ | Fragment VII_c_ cloned in front of *lacZ* gene of pDG1663 | This work |
| pDGP_c(F_VIIIc)_ | Fragment VIII_c_ cloned in front of *lacZ* gene of pDG1663 | This work |
| pDGP_r(F_Ir)_ | Fragment I_r_ cloned in front of *lacZ* gene of pDG1663 | This work |
| pDGP_r(F_IAr)_ | Fragment I_Ar_ cloned in front of *lacZ* gene of pDG1663 | This work |
| pDGP_r(F_IIIr)_ | Fragment III_r_ cloned in front of *lacZ* gene of pDG1663 | This work |
| pDGP_r(F_IVr)_ | Fragment IV_r_ cloned in front of *lacZ* gene of pDG1663 | This work |
| pDGP_r(F_Vr)_ | Fragment V_r_ cloned in front of *lacZ* gene of pDG1663 | This work |
| pDGP_r(F_VIIr)_ | Fragment VII_r_ cloned in front of *lacZ* gene of pDG1663 | This work |
| pDGP_r(F_VIIIr)_ | Fragment VIII_r_ cloned in front of *lacZ* gene of pDG1663 | This work |
| pRco_LS20_-His | *rco_LS20_* in the pET28b(+) vector | This work |
| pDP_spank_rco_LS20_-His | *rco*_LS20_-His cloned after the P_spank_ of the vector pDR110 | This work |
| *: D. Rudner, Department of Microbiology and Immunobiology, Harvard Medical School, 77 Avenue Louis Pasteur, Boston, MA 02115, USA | | |
|  |  |  |

**References**

3. Guerout-Fleury AM, Frandsen N, Stragier P (1996) Plasmids for ectopic integration in Bacillus subtilis. Gene 180: 57-61.

4. Itaya M, Sakaya N, Matsunaga S, Fujita K, Kaneko S (2006) Conjugational transfer kinetics of pLS20 between Bacillus subtilis in liquid medium. Biosci Biotechnol Biochem 70: 740-742. JST.JSTAGE/bbb/70.740 [pii].

5. Singh PK, Ramachandran G, Ramos-Ruiz R, Peiro-Pastor R, Abia D, Wu LJ, Meijer WJ (2013) Mobility of the Native Bacillus subtilis Conjugative Plasmid pLS20 Is Regulated by Intercellular Signaling. PLoS Genet 9: e1003892. 10.1371/journal.pgen.1003892 [doi];PGENETICS-D-13-01403 [pii].
