# Supplementary material for: A Complex Genetic Switch Involving Overlapping Divergent Promoters and DNA Looping Regulates Expression of Conjugation Genes of a Gram-positive Plasmid
Source: PLoS Genet. 2014 Oct 23;10(10):e1004733. doi: 10.1371/journal.pgen.1004733 (PMC4207663; doi:10.1371/journal.pgen.1004733)
Supplement: Table S3 — Oligonucleotides used. (DOCX) [file pgen.1004733.s006.docx]

| **Table S3. Oligonucleotides used** | | |
| --- | --- | --- |
| **Name** | **Sequence(5´-3´)** | **Purpose** |
| DIRECT | TCTCTATTGCCCACTTAT | Used in the primer extension to determine the position of P_c_ |
| INVERSE | TTCTAGTTCTTTTTACAC | Used in the primer extension to determine the position of P_c_ |
| MARKER1 | TTCTAGTTCTTTTTACAC | Used in the primer extension to determine the position of P_r_ |
| MARKER2 | ACGGTCTAGCGCTTACAAT | Used in the primer extension to determine the position of P_r_ |
| Prom28UPBam | cgcggatccTATACCACCTCGCAAAATAAACC | Used for the fragment I_c_ of inter-genic region between gene 28 and *rco_LS20_* |
| Prom28UPHind | ccccaagcttTATACCACCTCGCAAAATAAACC | Used for the fragment I_c_, I_r_, IV_c_, IV_r_, V_c_ , V_r_, VI_c_ and VI_r_ of inter-genic region between gene 28 and *rco_LS20_* |
| Xre prom_New | ttttaagcttGCACCAGCATCAAGTAACACTTGTTTCAG | Used for the fragment I_Ar_ of the intergenic region between gene 28 and *rco*_LS20_ |
| P28_Δ15 | tattaagcttGGGGCAAGTTCACACTAACTTTCACTGTG | Used for the fragment IV_c_ , VII_c_, IV_r_, and VII_r_ of inter-genic region between gene 28 and *rco_LS20_* |
| P28_Δ16 | tattaagcttGGCCCTTATCCTGTTTTTACTAAACTT | Used for the fragment V_c_, VIII_c_, V_r_, and VIII_r_ of alongwith inter-genic region between gene 28 and *rco_LS20_* |
| P28_Up_New | tattaagcttCCCTGTATACGGTCTAGCGCTTAC | Used for the fragment V_c_ and V_r_ of alongwith inter-genic region between gene 28 and *rco_LS20_* |
| OGR1 | tattaagcttCCGGTTAAAAATTTCACTGAAATAC | Used for the fragment VII_c_ and VII_r_ of inter-genic region between gene 28 and *rco_LS20_* |
| OGR2 | tattaagcttGTCAGTGAAAAAAATGCAGAATAAGG | Used for the fragment VIII_c_ and VIII_r_ of inter-genic region between gene 28 and *rco_LS20_* |
| OPKS8 | gggggtcgacGTCCTTTTTTAATTTCATGTATTC | Amplifying rco_LS20_for cloning in pET28b+ |
| OPKS14N | ggggccatggTGGGCAATAGAGAGCAATTTGATC | Amplifying rco_LS20_ for cloning in pET28b+vector |
| oGR34 | GTTCTTTTTACACAGAAATTGTTTG | Gel retardation assay |
| oGR35 | TTTTGATATAGCTCACAGTGAAAGT | Gel retardation assay |
| oGR36 | CCAAGTTGCAGCATGATTTTGAT | Gel retardation assay |
| oGR37 | CTAGAATTCAACTTTTGTTTTAACC | Gel retardation assay |
| oGR91 | AGCGCTTACAATTTTTCGCGTTTTT | Gel retardation assay |
| oGR92 | TTATTCTGCATTTTTTTCACTGACT | Gel retardation assay |
| oGR93 | GGTTTTTTGTTGTTAATCTCAAACA | Gel retardation assay |
| oGR94 | GACTACATTGTGATAGCACACTTTGA | Gel retardation assay |
| oGR95 | TACCAGTTAATTTAACCGTATGTAT | Gel retardation assay |
| oGR96 | ATTTTTAACCGGTTTTTTTCACTGAG | Gel retardation assay |
| oGR97 | ATTTTTCGCGTTTTTTTTACT**T**AGTGA**CCCCC**A | Gel retardation assay |
| oGR98 | TTATTCTGCAT**GGGGG**TCACTGACTCTTTCACTAA | Gel retardation assay |
| oGR99 | TTTTGCTAGCGTAAGGATGG**A**GGAATTTTCTTGCG | Gel retardation assay |
| oGR135 | TTTTTACACAGAAATTGTTTGAGATTAACAA**GGATCC**AAAAAACCTTATTCTGCA | PCR overlapping to add 5 bps to the intergenic region between genes *rco_LS20_* and 28. |
| oGR136 | **GGATCC**TTGTTAATCTCAAACAATTTCTGTGTAAAAAGAACTA**GAATTC**AAC | PCR overlapping to add 5 bps to the inter-genic region between genes *rco_LS20_* and 28. |
| oGR138 | GCCTCTCTTTTAAAGCTAAAATGATGTATG | Circularization assay |
| 5´- overhang sequences are indicated in lower case and restriction sites are underlined; Mutations or additions marked in bold and underlined | | |
